# Arrhythmogenic Right Ventricular Cardiomyopathy in Pediatric Patients: An Important but Underrecognized Clinical Entity

**DOI:** 10.3389/fped.2021.750916

**Published:** 2021-12-02

**Authors:** Anneline S. J. M. te Riele, Cynthia A. James, Hugh Calkins, Adalena Tsatsopoulou

**Affiliations:** ^1^Division Heart & Lungs, Department of Cardiology, University Medical Center Utrecht, Utrecht University, Utrecht, Netherlands; ^2^Netherlands Heart Institute, Utrecht, Netherlands; ^3^Division of Cardiology, Department of Medicine, Johns Hopkins Hospital, Baltimore, MD, United States; ^4^Unit of Inherited and Rare Cardiovascular Diseases, Onassis Cardiac Surgery Center, Athens, Greece

**Keywords:** arrhythmogenic (right ventricular) cardiomyopathy, natural history, management, children, adolescent, pediatric, naxos disease

## Abstract

Arrhythmogenic right ventricular cardiomyopathy (ARVC) is an inherited cardiomyopathy characterized by fibrofatty infiltration of predominantly the right ventricular (RV) myocardium. Affected patients typically present as young adults with hemodynamically stable ventricular tachycardia, although pediatric cases are increasingly recognized. These young subjects often have a more severe phenotype with a high risk of sudden cardiac death (SCD) and progression toward heart failure. Diagnosis of ARVC is made by combining multiple sources of information as prescribed by the consensus-based Task Force Criteria. The description of Naxos disease, a fully penetrant autosomal recessive disorder that is associated with ARVC and a cutaneous phenotype of palmoplantar keratoderma and wooly hair facilitated the identification of the genetic cause of ARVC. At present, approximately 60% of patients are found to carry a pathogenic variant in one of five genes associated with the cardiac desmosome. The incomplete penetrance and variable expressivity of these variants however implies an important role for environmental factors, of which participation in endurance exercise is a strong risk factor. Since there currently is no definite cure for ARVC, disease management is directed toward symptom reduction, delay of disease progression, and prevention of SCD. This clinically focused review describes the spectrum of ARVC among children and adolescents, the genetic architecture underlying this disease, the cardio-cutaneous syndromes that led to its identification, and current diagnostic and therapeutic strategies in pediatric ARVC subjects.

## Introduction

**Figure 1:**
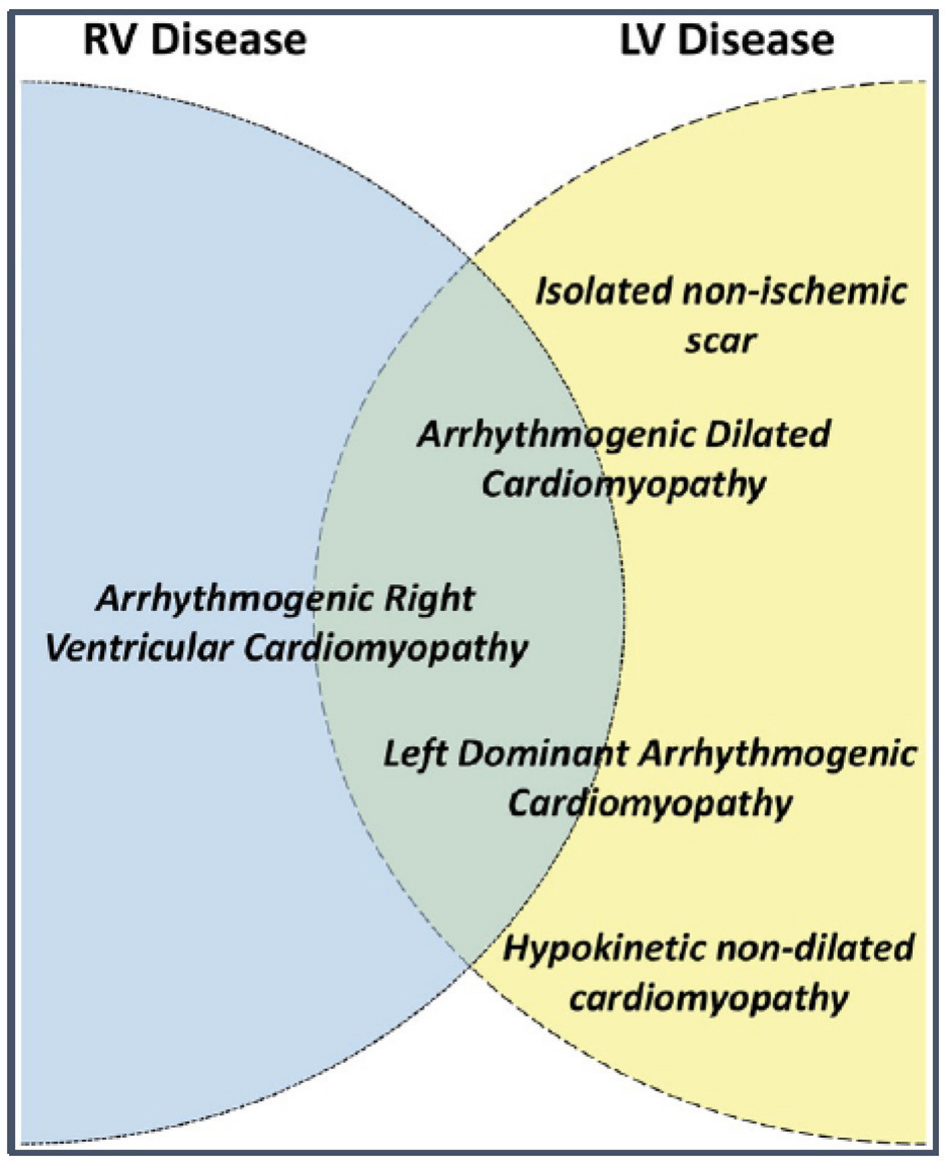
Terms used to describe different arrhythmogenic cardiomyopathy phenotypes and their possible relationship to left (LV) and right ventricular (RV) disease. Source: (1), obtained with permission.

Arrhythmogenic right ventricular cardiomyopathy (ARVC) is a heritable condition of the heart and is part of the phenotypic spectrum of arrhythmogenic cardiomyopathies (ACM) that includes right-dominant (ARVC), left-dominant (ALVC) and biventricular arrhythmogenic cardiomyopathy (Figure 1) (1, 2). Our manuscript focuses on the right-dominant subform of pediatric ACM (i.e., ARVC); a description of left-dominant and biventricular ACM in pediatric patients can be found in the same issue of this journal (reference). ARVC is characterized by potentially severe ventricular arrhythmias and structural alterations of the ventricular myocardium, which are identified by macro- and microscopic pathological examination and/or abnormal cardiac imaging (1). While right ventricular (RV) structural abnormalities have been predominantly reported, advanced imaging techniques reveal increased incidence of left ventricular (LV) or biventricular involvement (3–5). A sudden death that occurred during exercise in a young doctor, who was previously noted to have episodes of sustained ventricular tachycardia (VT) of RV origin, was the “case 0” of the fundamental report of ARVC as a cause of juvenile sudden death (6, 7). Indeed, although the estimated prevalence of ARVC in the general population is only 1:5,000, it represents one of the most common causes of juvenile sudden death (8).

Since the initial disease description by Marcus and colleagues in 1982, most studied ARVC cohorts constituted mainly of adults (3). Arrhythmia-related episodes characterize affected patients, who commonly present at late adolescence or young adulthood. In subsequent years, familial occurrence of ARVC was recognized (9, 10), and pediatric cases were increasingly reported. These young patients often present with a high risk of severe ventricular arrhythmias or sudden cardiac death (11), underscoring the need for early disease detection. Nonetheless, reduced penetrance and variable expressivity in familial ARVC, complicated linkage studies during the early genotyping attempts (7). A breakthrough came with the identification of Naxos syndrome, a fully penetrant autosomal recessive disorder associating ARVC with a cutaneous phenotype of palmoplantar keratoderma and wooly hair that characterizes the patient from infancy. A pathogenic variant in the desmosomal protein junctional plakoglobin (*JUP*) was identified in Naxos disease and was the first causative gene for ARVC (12). Subsequently, pathogenic variants in other desmosomal proteins were related to ARVC. As a result, ARVC is currently considered a heritable desmosomal disorder and more than 60% of probands are now found to harbor a pathogenic desmosomal variant. Yet, non-desmosomal genes are increasingly identified among those with biventricular or left-dominant disease (13).

Although disease evolution and prognosis have been studied in large cohorts of index patients and family members (14), the question of when and how the disease manifests in children has not yet been elucidated. Few cohorts and sporadic cases of children with ARVC have been reported. A systematic follow-up of asymptomatic children carrying a pathogenic genetic variant will provide important information on disease initiation and uncover the earliest signs on how ARVC evolves during childhood.

This clinically focused review describes the spectrum of ARVC among children and adolescents, the genetic architecture underlying this disease, and the current diagnostic and therapeutic strategies in pediatric ARVC subjects.

## Diagnosis

The diagnosis of ARVC may be challenging, as no single modality is sufficiently sensitive or specific to serve as the gold standard for ARVC diagnosis. As such, multiple sources of diagnostic information are combined in a complex set of criteria determined by a Task Force in 1994, which were subsequently modified in 2010 (15). These so-called “Task Force Criteria” (TFC) include major and minor criteria encompassing structural, histologic, electrocardiographic (i.e., depolarization and repolarization), arrhythmic, and family history findings (Table 1). Quantitative TFC criteria were derived from a comparison of 108 ARVC probands to healthy controls, in which cut-offs for major criteria were chosen to achieve 95% specificity. Cut-offs with high specificity invariably result in lower sensitivity, which ranged from 17–58% in a recent validation study (16). In contrast, minor criteria have higher sensitivity (up to 82%) but consequently lower specificity (as low as 67%) (16). Overall, 2 major, 1 major and 2 minor, or 4 minor criteria are required for diagnosis.

**Table 1:** Diagnostic Task Force Criteria for arrhythmogenic right ventricular cardiomyopathy.

**I. Structural/functional assessment**	
**Major**	**2D Echocardiography:**
	•Regional RV akinesia, dyskinesia, or aneurysm •*and* 1 of the following at end diastole: ° PLAX RVOT ≥ 32 mm or PLAX/BSA ≥ 19 mm/m^2^ ° PSAX RVOT ≥ 36 mm or PSAX/BSA ≥ 21 mm/m^2^ ° Fractional area change ≤ 33%
	**CMR:**
	•Regional RV akinesia or dyskinesia or dyssynchronous contraction •*and* 1 of the following: ° RV EDV/BSA ≥ 110 mL/m^2^ (male) or ≥ 100 mL/m^2^ (female) ° RVEF ≤ 40%
	**RV angiography:**
	Regional RV akinesia, dyskinesia, or aneurysm
**Minor**	**2D Echocardiography:**
	•Regional RV akinesia, dyskinesia, or aneurysm •*and* 1 of the following at end diastole: ° PLAX RVOT ≥ 29 mm or PLAX/BSA ≥ 16 mm/m^2^ ° PSAX RVOT ≥ 32 mm or PSAX/BSA ≥ 18 mm/m^2^ ° Fractional area change ≤ 40%
	**CMR:**
	•Regional RV akinesia or dyskinesia or dyssynchronous contraction •*and* 1 of the following (end diastole): ° RV EDV/BSA ≥ 100 mL/m^2^ (male) or ≥ 90 mL/m^2^ (female) ° RVEF ≤ 45%
**II. Tissue characterization**	
**Major**	Residual myocytes <60% by morphometric analysis (or <50% if estimated), with fibrous replacement of the RV free wall myocardium in ≥1 sample, with or without fatty replacement of tissue on endomyocardial biopsy.
**Minor**	Residual myocytes 60–75% by morphometric analysis (or 50–65% if estimated), with fibrous replacement of the RV free wall myocardium in ≥1 sample, with or without fatty replacement of tissue on endomyocardial biopsy.
**III. Repolarization abnormalities**	
**Major**	Inverted T-waves in leads V1, V2, and V3 or beyond, in individuals >14 years of age (in absence of complete RBBB QRS ≥120 ms).
**Minor**	•Inverted T-waves in leads V1 and V2, in individuals >14 years of age (in absence of complete RBBB) or in V4, V5, or V6. •Inverted T-waves in leads V1, V2, V3, and V4 in individuals >14 years of age in the presence of complete RBBB.
**IV. Depolarization abnormalities**	
**Major**	Epsilon wave (reproducible low-amplitude signals between end of QRS complete to onset of the T-wave) in V1–3.
**Minor**	•Late potentials by SAECG in ≥1 of 3 parameters in absence of a QRS of ≥110 ms on standard ECG: ° Filtered QRS duration ≥114 ms ° Duration of terminal QRS <40 μV ≥38 ms ° Root-mean-square voltage of terminal 40 ms ≤ 20 μV •Terminal activation duration of QRS ≥55 ms, measured from the nadir of the S-wave to the end of the QRS, including R', in V1, V2, or V3, in absence of complete RBBB.
**V. Arrhythmias**	
**Major**	Non-sustained or sustained VT of LBBB morphology with superior axis.
**Minor**	•Non-sustained or sustained VT of RVOT configuration, LBBB morphology with inferior axis or with unknown axis. •>500 PVCs per 24 h on Holter monitoring
**VI. Family history**	
**Major**	•First-degree relative with ARVC confirmed by TFC •First-degree relative with ARVC confirmed pathologically at autopsy or surgery •Identification of a pathogenic variant categorized as associated or probably associated with ARVC in the patient under evaluation
**Minor**	•First-degree relative with ARVC history not possible to confirm by TFC •First-degree relative with SCD <35 years of age due to suspected ARVC •Second-degree relative with ARVC confirmed by TFC or pathologically

*ARVC, arrhythmogenic right ventricular cardiomyopathy; BSA, body surface area; CMR, cardiac magnetic resonance; LBBB, left bundle branch block; EDV, end-diastolic volume; PVC, premature ventricular complex; RBBB, right bundle branch block; PLAX, parasternal long axis; PSAX, parasternal short axis; RV, right ventricular; RVEF, right ventricular ejection fraction; RVOT, right ventricular outflow tract; SAECG, signal-averaged electrocardiogram; SCD, sudden cardiac death; TFC, Task Force Criteria; VT, ventricular tachycardia*.

**Figure 2:**
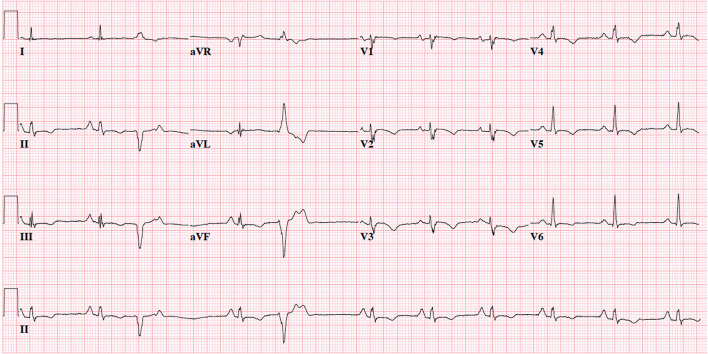
12-Lead electrocardiogram of 19-year old female with advanced ARVC. Note normal sinus rhythm with frequent premature ventricular complexes with superior axis morphology, voltage criteria for right atrial enlargement, borderline prolonged QRS duration (QRS 0.10s) with fractionated upstroke in V1-3 (“terminal activation duration” >55 ms), and negative T-waves in V1-6 and inferior leads. This patient would fulfill 1 major (T-wave inversion V1–V3 and beyond) and 1 minor criterion (prolonged terminal activation duration) for ARVC. ECG settings: 25 mm/mV; 10 mm/mV; 150 Hz.

**Figure 3:**
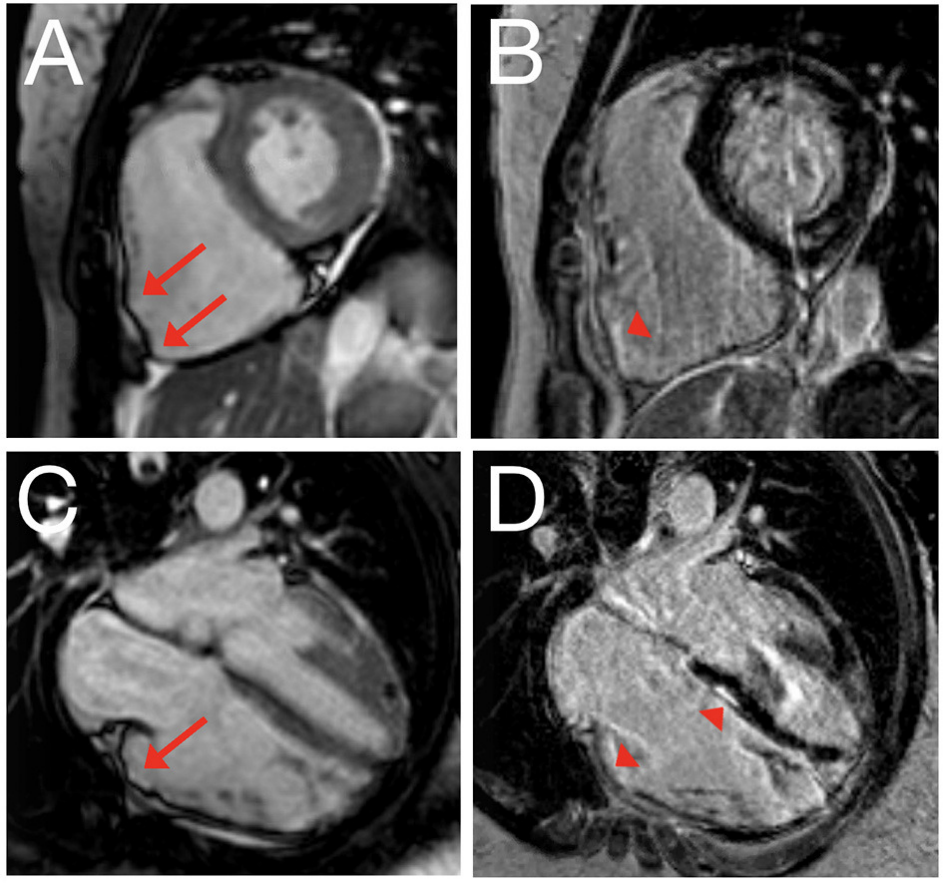
Cardiac magnetic resonance imaging of 22-year old female with ARVC. Still image of cine clip for short axis **(A)** and long axis **(C)** view, with corresponding delayed enhancement images obtained using phase-sensitive inversion recovery in short axis **(B)** and long axis **(D)** view. Red arrows denote dyskinetic segments with outward bulging in systole; arrowheads denote corresponding delayed enhancement.

For those involved in the care of pediatric ARVC patients, it is important to recognize that the 2010 TFC were derived using a predominantly adult population (mean 38 ± 13 years of age): in fact, only 9 of 108 (8%) probands in the original TFC document were diagnosed between 12 and 18 years of age (15). As such, extrapolation of the TFC for use in pediatric cases should be considered experimental (17). The most important concern for TFC implementation during childhood probably rises for ECG criteria: it is widely accepted that right precordial T-wave inversion V1-3 (major criterion for ARVC diagnosis) is a normal finding in children before puberty (18), and therefore the TFC disregard T-wave inversion as diagnostic criterion prior to the age of 14 years. In addition, epsilon waves (also a major criterion for ARVC diagnosis) are often seen in advanced ARVC cases and are likely to be extremely rare in children and adolescents (19). This potentially results in two major criteria with very little (if any) utility in pediatric patients. A typical ECG for an ARVC patient is shown in Figure 2. Another equally important concern is that echocardiographic and cardiac magnetic resonance (CMR) criteria are not validated in children. Indeed, the revised TFC determined imaging cut-offs based on a cohort with a mean age of 60 years. Since that time, several reports showed that RV end-diastolic volume decreases on average 4% per decade in healthy subjects, which is not accounted for by adjusting for body surface area (20). This suggests that the TFC may be too sensitive in pediatric patients. A typical CMR for an ARVC patient is shown in Figure 3.

Regardless of these limitations, several studies have evaluated the performance of the revised TFC in pediatric or adolescent cohorts. In 2015, Etoom et al. showed that conventional CMR criteria were the strongest contributor to TFC fulfillment in children with ARVC, with almost half of the study population relying on CMR criteria for diagnosis (21). While fatty infiltration and delayed enhancement were rare in this Canadian cohort, a subsequent case series by Slesnick et al. showed that CMR can be highly valuable in determining fibrofatty replacement in pediatric ARVC subjects (22). Similar findings were observed by Steinmetz et al. who showed that any TFC fulfillment on imaging was associated with definite ARVC diagnosis in children with an odds ratio of 8.68 (23). While these studies suggest an important role for imaging in pediatric ARVC evaluation, the relatively low prevalence of imaging criteria (particularly using echocardiography) and possibly false-positive results in an important subset of patients are cause for concern (24). Several authors have therefore called for the determination of pediatric-specific diagnostic criteria (25, 26). Until the present time, however, the 2010 TFC remain the clinical gold standard for ARVC evaluation in childhood and adolescence. This seems an appropriate decision, as the pathognomonic criteria for ARVC diagnosis in adults are also valid for young people (27), and the clinical characteristics and outcomes are similar between pediatric and adult patients with a definite ARVC diagnosis as per TFC (11).

## Genetics

### Genetic Architecture of ARVC

As described above, ARVC is conventionally considered a disease of the cardiac desmosome with autosomal dominant inheritance and age-related reduced penetrance. Pathogenicity of genetic variants in ARVC evaluation is typically attributed based on the American Society of Clinical Genetics (ACMG) criteria; where class 4 (likely pathogenic) and class 5 (pathogenic) variants are considered to be associated with disease (28). In contemporary cohorts, up to two-thirds of patients with definite ARVC per the 2010 TFC have a pathogenic or likely pathogenic (P/LP) variant in one of 5 genes encoding cardiac desmosome proteins (*PKP2, DSP, DSG2, DSP*, and *JUP*) (14, 29). Well-characterized variants in *TMEM43* and *PLN* also contribute to ARVC, particularly in specific geographic populations (30, 31). Heterozygous truncating and splice P/LP variants in *PKP2* are the most common genetic cause of ARVC (29). *DSP, DSG2*, and *PLN* variants are seen more frequently in those with biventricular or left-dominant disease (30, 32–34).

**Figure 4:**
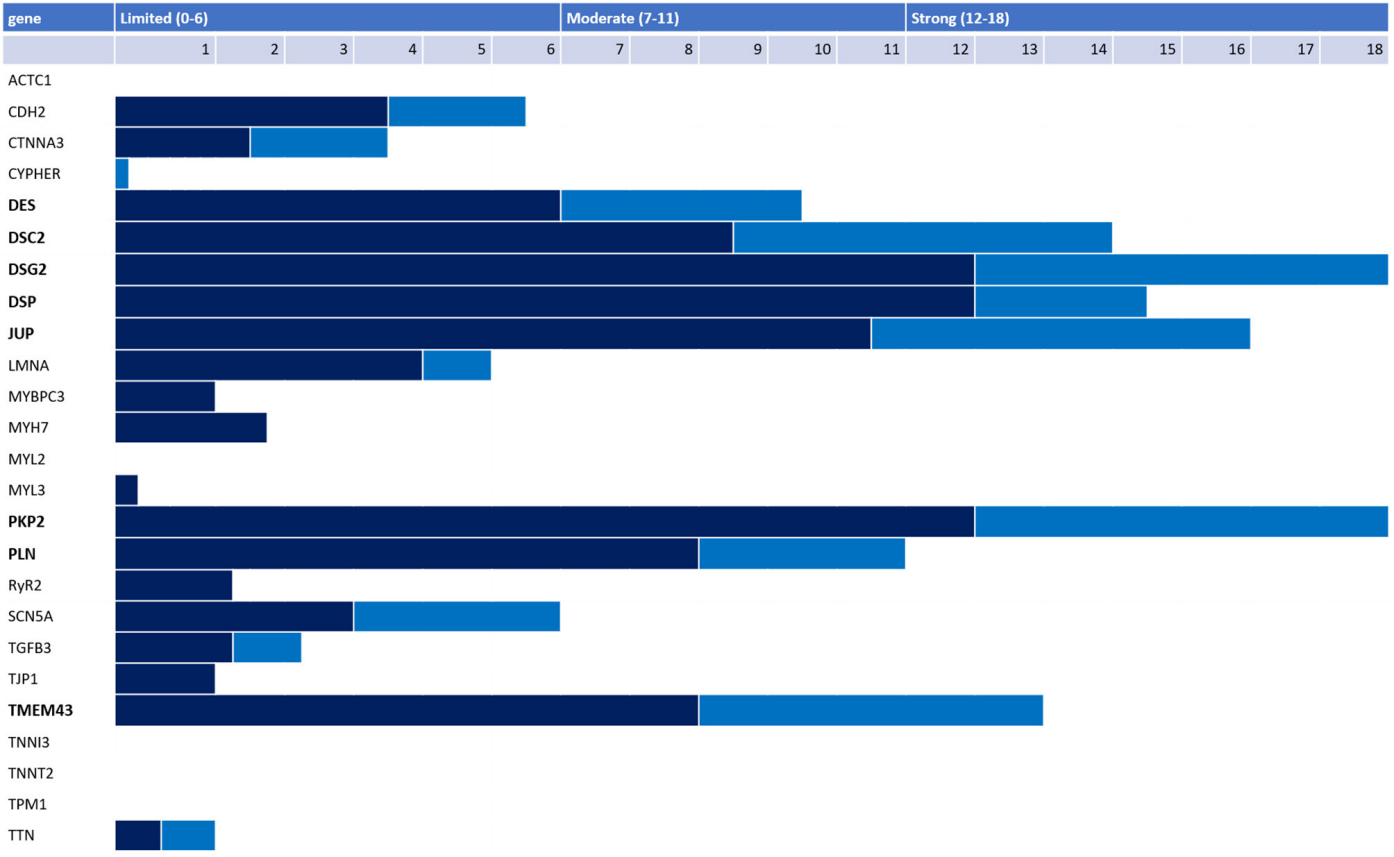
Adjudication of 26 genes reported as ARVC-causing in the literature by an international expert panel. Genetic (dark blue) and experimental (light blue) evidence and final classification 26 genes reported in the literature as associated with ARVC per the ClinGen framework. Only 8 genes had strong or moderate evidence for ARVC causality (bold).

Numerous additional ARVC genes have also been proposed, with uneven quantity and quality of evidence underpinning the ARVC-gene association. Recently, a panel of international experts conducted a rigorous reappraisal of 26 ARVC genes reported in the literature using the semi-quantitative ClinGen framework and found only eight genes had definitive (*PKP2, DSP, DSG2, DSC2, JUP, TMEM43)* or moderate (*PLN, DES*) evidence for causing ARVC (Figure 4) (35). Notably, *RYR2* was disqualified as an ARVC gene with the investigators reporting that patients and model systems in the literature had catecholaminergic polymorphic ventricular tachycardia (CPVT), not ARVC. Some genes with only limited evidence are relatively newly characterized and more evidence of ARVC causality may accrue over time. Nonetheless, based on current evidence available, the authors suggested that P/LP variants in only *PKP2, DSP, DSG2, DSC2, JUP, TMEM43, PLN*, and *DES* should be used to assign a major genetics criterion when applying the 2010 TFC. Ensuring only genes with substantial evidence of ARVC causality are used to assign the major genetics criterion in the TFC is particularly important for pediatric patients being evaluated for ARVC: they may rely more on this criterion for diagnosis as the TFC disregard right precordial T-wave inversion as a diagnostic criterion before age 14 (18).

Young ARVC patients are particularly likely to harbor one or more P/LP variants. In a cohort of American and Dutch pediatric patients (defined as diagnosis before age 18 or symptomatic presentation of a proband before age 18), 80% had a P/LP variant, significantly more than the 60% of 427 patients presenting as adults (11). Similarly, in a combined cohort of pediatric patients with classic ARVC, biventricular disease, and left-dominant arrhythmogenic cardiomyopathy, 75% of patients with ARVC had at least one P/LP desmosomal variant (36). In the aggregate, genotype positive patients (e.g., harboring one or more P/LP variants) present symptomatically 4 years earlier than gene-elusive probands (14).

### Genotype-Phenotype Associations

Broadly, gene-elusive ARVC patients have a similar clinical course to patients with a single P/LP desmosomal variant: a similar proportion develop symptoms, have similar survival free from sustained ventricular arrhythmias, or require transplant (14). Nonetheless, useful data linking genotype to clinical outcomes is emerging. Of particular importance in pediatrics, patients with more than one P/LP variant (including homozygous, compound heterozygous, or di- and tri-genic variants) have younger onset, worse clinical outcomes, and may have an atypical phenotype (37). In a combined US and Dutch cohort of 577 patients with P/LP variants in the desmosomal genes, *TMEM43*, or *PLN*, the 4% of patients with more than one variant had significantly earlier occurrence of sustained VT (mean age 28 ± 12 years), worse survival free from a first sustained ventricular arrhythmia, and more frequent LV dysfunction (29%), heart failure (19%) and cardiac transplantation (9%) when compared with those with only one P/LP variant (32). In a cohort of 32 ARVC patients with pediatric onset described by DeWitt et al., multiple potentially pathogenic variants (P/LP and variants of uncertain significance [VUS]) were identified in 9 (28%) patients including 6/9 (67%) with biventricular disease (36). Notably, the presence of multiple P/LP *PKP2* variants with at least one on each allele (homozygous or compound heterozygous) seems to herald poor outcomes including pediatric sudden cardiac death and transplant at a young age (36, 38, 39). The presence of loss of function *PKP2* variants on each allele (homozygous or compound heterozygous null alleles) may be particularly devastating. There are multiple reports of infants/fetuses with congenital heart disease and poor neonatal outcomes with this genotype (40, 41), consistent with the requirement of plakophilin-2 for cardiac development (42).

Investigators have considered whether genotype is a useful predictor of arrhythmic risk in ARVC, but with only limited success. As noted above, patients with more than one P/LP variant have worse arrhythmic outcomes. Various papers have suggested that particular genes are associated with higher arrhythmic risk, but most of these assertions have not been replicated. Furthermore, in two statistical models developed recently for individualized risk prediction for incident sustained ventricular arrhythmias and rapid sustained ventricular arrhythmias for ARVC patients, genotype was not a significant predictor and is therefore not included in either model (www.arvcrisk.com) (43, 44). This is notable especially because the multicenter international cohorts included to derive the models were the largest cohort of ARVC patients ever assembled. One exception to the limited utility of genotype in predicting arrhythmic risk is the *TMEM43* c.1073C>T; p.Ser358Leu variant which was initially identified in a large number of patients and families from Newfoundland. This variant is associated with a highly penetrant and arrhythmogenic subtype of ARVC (45, 46). Risk of sudden cardiac death from ventricular arrhythmias is particularly high in males. As such, the literature suggests that genotype predicts arrhythmic risk in ARVC less well than demographic and clinical predictors.

**Figure 5:**
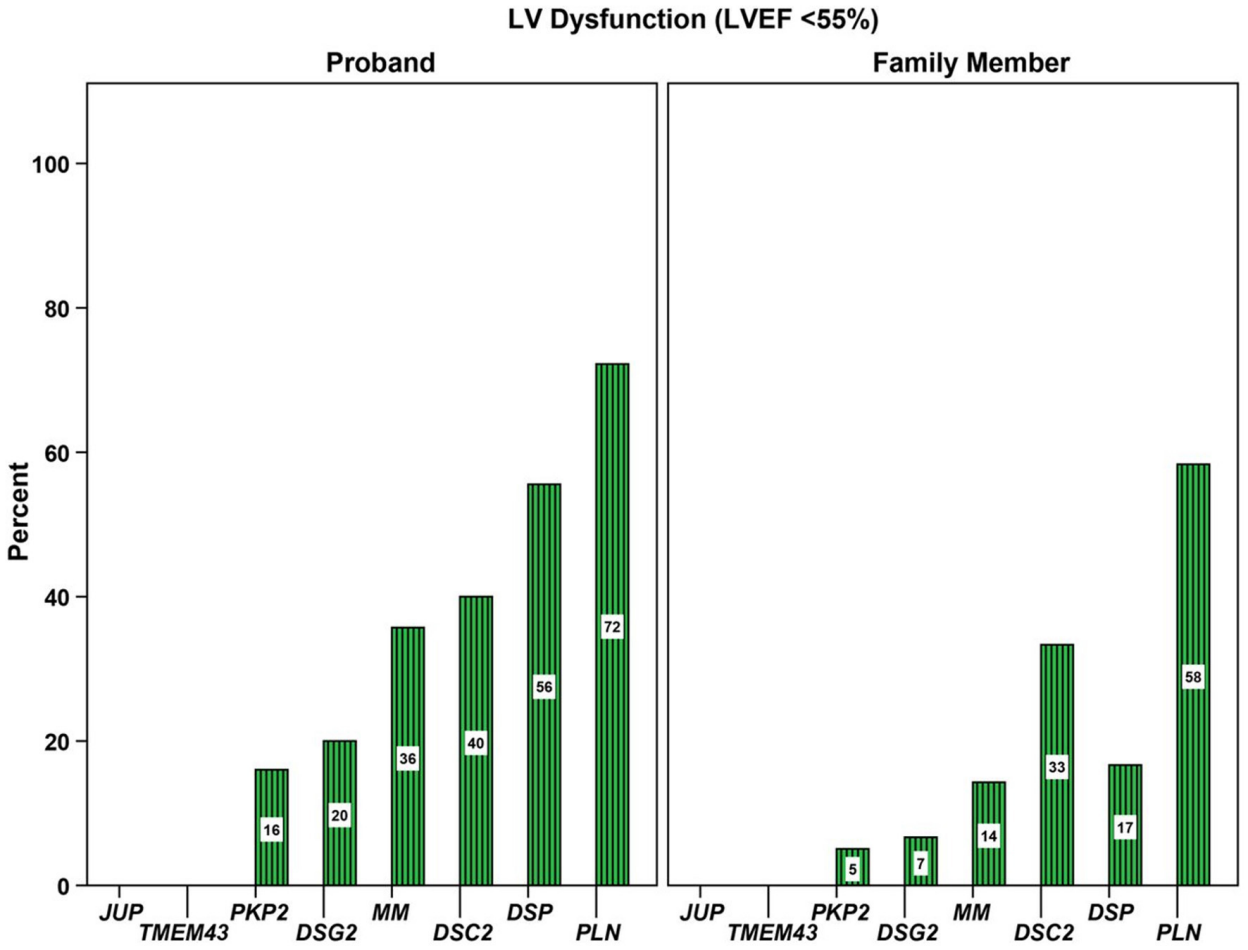
Association of genotype with prevalence of left ventricular (LV) dysfunction. Proportion of 577 gene-positive probands and family members with LV dysfunction stratified by gene with a pathogenic or likely pathogenic variant. LV, left ventricular; MM, more than one P/LP variant, including compound heterozygote, homozygote, and digenic patients. Source: (32), obtained with permission.

In contrast, there is substantial evidence that the extent of LV involvement and heart failure is associated with genotype. A large study in 577 ARVC probands and family members with P/LP variants from North America and The Netherlands showed that prevalence of left ventricular dysfunction and heart failure varied substantially by genotype (32). LV dysfunction (defined as LV ejection fraction <55%) was seen in 78 (14%) patients while 28 (5%) experienced clinically-recognized congestive heart failure during follow-up. As shown in Figure 5, *PKP2* carriers were least likely to have LV dysfunction (9%), whereas those with a P/LP *DSP* variant had significantly more frequent LV dysfunction (40%) and heart failure (13%). *PLN* variant carriers presented at a significantly older age yet had worse long-term prognosis, with more LV dysfunction and heart failure. The observation that P/LP variants in *DSP* and *PLN* disproportionately affect the LV has been replicated in numerous studies including in pediatric patients with *DSP* variants (36). Furthermore, as discussed earlier, multiple P/LP variants heralded worse outcomes, tripling of the prevalence of heart failure (32). The latter was also found in a British study, in which four of eight patients who underwent transplantation or died due to heart failure had multiple genetic variants (47). It is also important to realize that the influence of founder variants is significant: for example, the homozygous p.F531C variant in *DSG2* was associated with a fully penetrant heart failure phenotype in a Chinese cohort (34), while the *TMEM43* p.S358L variant in addition to conveying a high risk of sudden cardiac death is associated with LV dysfunction (31).

Finally, there is increasing evidence that ARVC presenting with chest pain and myocardial enzyme release in the setting of normal coronary arteries ('hot phase') is particularly common in patients with DSP P/LP variants (48–50). These myocarditis-like episodes of acute myocardial injury are particularly common in pediatric ARVC, may recur, and can be familial. Therefore, recurrent myocarditis in a child, or pediatric myocarditis in the setting of a concerning family history should prompt consideration for genetic testing and for evaluation for ARVC.

### Genetic Testing

Genetic testing is recommended for patients with ARVC to confirm the diagnosis, inform management, and inform cascade testing of the family (2, 51). More information on cascade genetic testing can be found below. A multidisciplinary team approach including cardiology, pathology, genetics, and genetic counseling is recommended and valued by patients (2). Best practices for genetic testing for ARVC have been reviewed elsewhere (2, 52), but several aspects that are particularly relevant to the pediatric population are described below and summarized in Table 2.

**Table 2:** Pediatric genetic testing overview.

	**Who to test?**	**When to test?**	**How to test?**	**Why to test?**
**First test in family**	- Youngest onset - Worst phenotype	- After complete cardiovascular evaluation - After constructing pedigree	- Full coverage of ARVC genes - Test that includes detection of copy number variants - Large panel a reasonable choice - With psychosocial support and multidisciplinary team	- To inform cascade screening of family. - Genotype-guided care
**Cascade testing**	- First degree relatives ≥ age 10, younger testing reasonable^a^, ^b^ - Families with a P/LP variant	- In conjunction with baseline cardiovascular screening	- Targeted sequencing of family variant(s) - With psychosocial support and genetic counseling	- Determine need for longitudinal cardiovascular screening - Inform decisions about exercise

*References to substantiate statement:*

a *= (51);*

b *= (2)*.

*P/LP, pathogenic or likely pathogenic variant as assessed by criteria published by the American College of Medical Genetics and Genomics and Association for Molecular Pathology (28)*.

## Lessons Learned From Naxos Disease

It was as early as 1986 when the first observation of an ARVC phenotype associated with palmoplantar keratoderma (PPK) and wooly hair (WH) was published. The syndrome was named “Naxos disease” after the Aegean island where it was initially observed, and it was reported under this name in the World Congress on Cardiomyopathies in Warsaw in 1993.

Over the years that followed, Naxos disease was mapped to chromosome 17q21 and the causative pathogenic variant was identified: a 2 base-pair deletion in the desmosomal *JUP* gene (*Pk2157del2*TG), truncating the C-terminal of the protein (53). A decade after the first publication, a similar phenotype was reported by Rao et al. and by Carvajal et al. in patients from India (1996) and Ecuador (1998), respectively (54). The cutaneous phenotype consisting of PPK and WH seemed identical to that of Naxos syndrome, while the cardiomyopathy presented with predominant LV involvement diagnosed as dilated cardiomyopathy. Molecular genetic investigations in the families from Ecuador revealed a recessive variant in *DSP* (55). In a later report on an Ecuadorian patient, the name of Carvajal syndrome was used for the first time (56). It is worth mentioning, however, that Hamill et al. had reported on a syndromic phenotype of dilated cardiomyopathy with arrhythmias and ectodermal dysplasia in three unrelated young toddlers as early as 1988 (57). The children presented with episodes of recurrent VT by the age of 18 months, while chest pain and congestive heart failure developed by the 3rd year of life. Severe biventricular involvement with fibrosis and moderate inflammatory infiltrates was detected. Alopecia preceded the cardiac phenotype, while lesions of PPK, nail and dental anomalies were noted despite their young age.

The homozygous pathogenic *JUP* variant is identified in other Aegean islands as well as in Turkey (58, 59). The prevalence of Naxos disease in Greek islands reaches 1:1,000 (60). Over the years, an increasing number of families with the phenotype of WH, PPK and ARVC/ACM left-dominant arrhythmogenic cardiomyopathy have been reported. Most of these reports concern pathogenic *DSP* variants (54, 61, 62). It is important to note that patients with Naxos or Carvajal disease are rare (outside of endemic regions), and that they present with a distinct cutaneous and cardiac phenotype. Regardless, many lessons were learned based on Naxos disease patients, as described below.

### The Cutaneous Phenotype

In patients with Naxos disease, WH is apparent from birth. Interestingly, observant members of affected families have mentioned it as a harbinger of a severe heart disease (personal communication). In the largest reported series of pathogenic *DSP* variant carriers, WH was shown to have high specificity in detecting subjects who will develop a cardiomyopathy (62). In some patients with *JUP*/homozygous pathogenic variants (or biallelic), sparse WH or even alopecia were reported. Alopecia has been related mostly to *DSP* variants (63).

**Figure 6:**
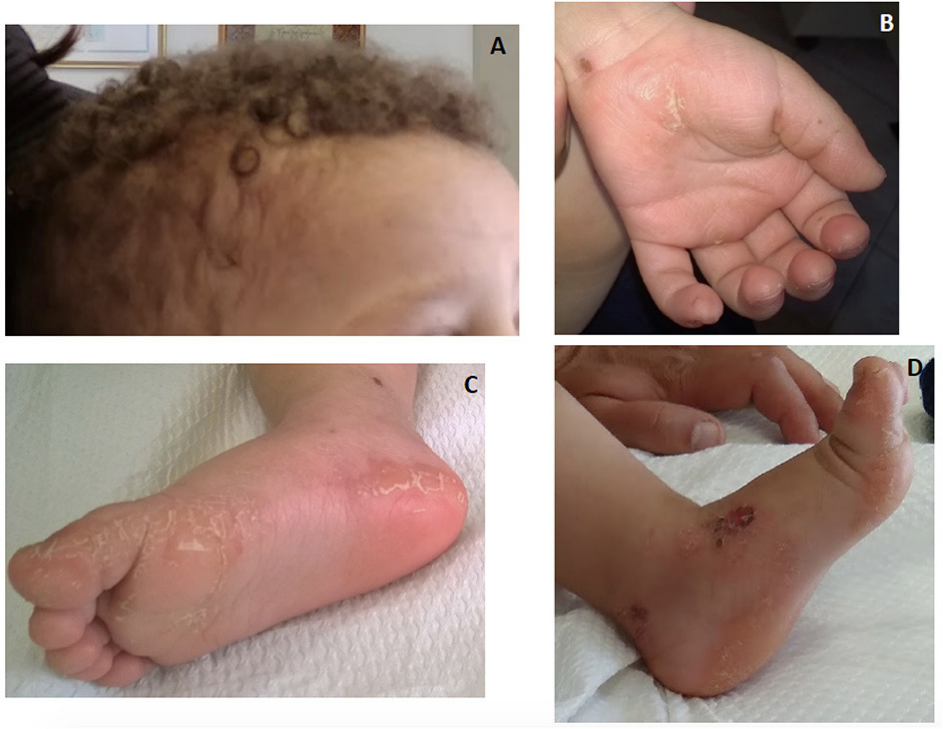
An 18-month-old boy with Naxos disease, homozygous for a pathogenic *JUP* variant. Wooly hair apparent from birth **(A)** and mild hyperkeratosis on palmar and more prominent on plantar areas **(B)** and **(C)**. Eczematous lesions and fragile skin with erosion on the dorsal foot area **(D)**.

Keratotic lesions on the palms and soles generally develop during the second year of life when the child is using hands and feet (60, 63). In early infancy, eczematous lesions, fragile skin or even erosion and ulcers have been observed on the perioral and sacral areas or dorsal surfaces of the hands and legs (Figure 6). Nail dystrophy and dental anomalies may be part of the phenotype particularly of pathogenic *DSP* variants (61, 64). Pemphigus-like vesicular lesions on palms, soles and knees have been reported as well (65). Of note, neonatal lethal epidermolysis has been reported in both *JUP* and *DSP* homozygous variants (63, 66, 67).

Immunohistology of non-lesional skin on the forearm in pathogenic *DSP* variant carriers revealed that desmoplakin is irregularly localized in the basal layers of epidermis instead of the membranous distribution observed in normal skin. The same, albeit to a lesser extent, is observed for plakoglobin. In areas of clinically “normal” skin, reduced expression of connexin43 is also observed (62). Expression of desmosomal proteins in basal layers of epidermis are more representative of those expressed in myocardium.

### The Cardiac Phenotype

**Figure 7:**
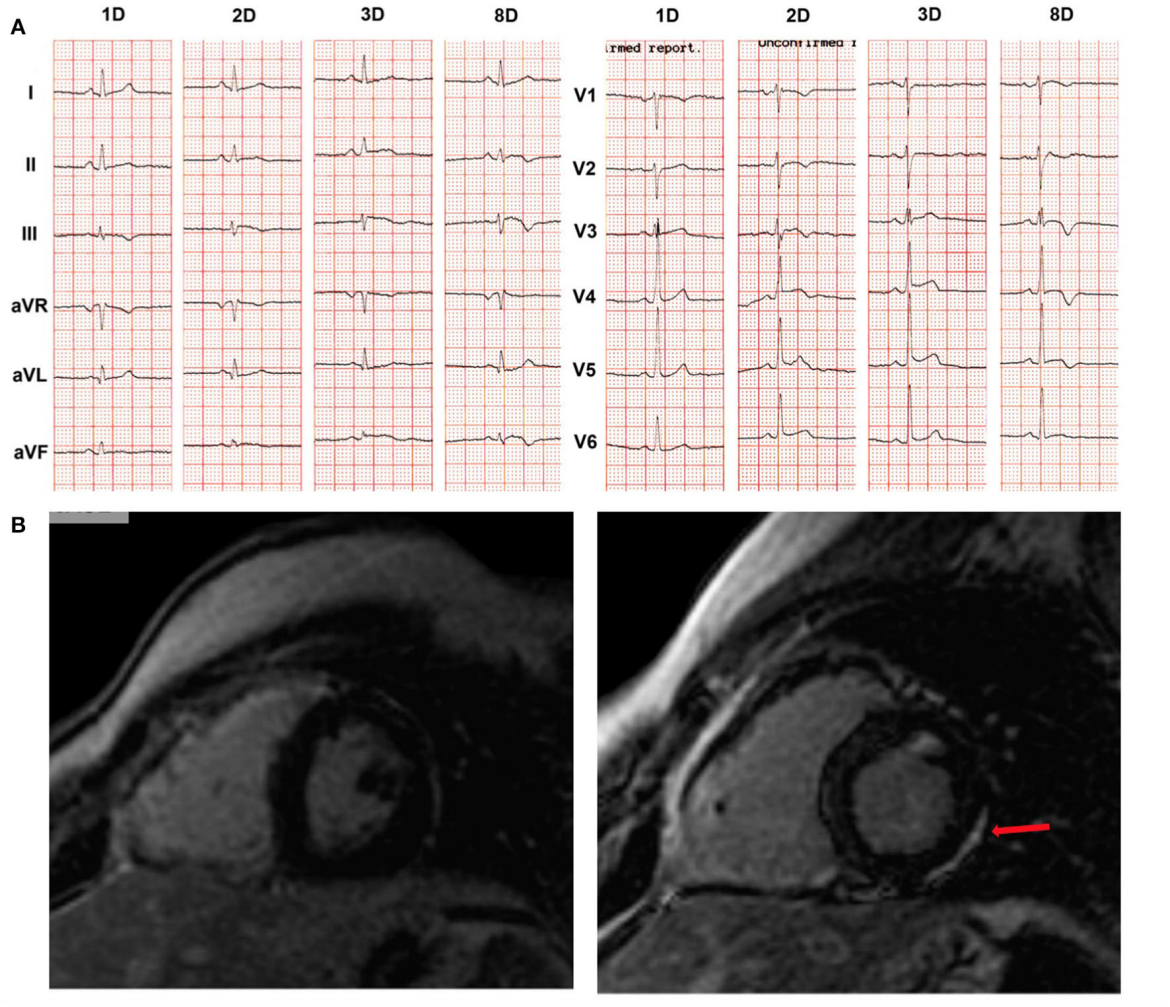
Example of a boy with Naxos disease, homozygous for a pathogenic *JUP* variant, on regular follow-up since the age of 1 year old. He was asymptomatic until the age of 14 years when he was admitted with chest pain, increased troponin-I levels up to 20 μg/L (normal values: 0.04 μg/L), complex ventricular extrasystoles and changes on 12-lead ECG. **(A)** ECG recordings in 25 mm/s, 10 mm/mV, at the first (1D), second (2D), third (3D) and eighth (8D) days of hospitalization. Newly developed repolarization changes are observed. **(B)** Phase-sensitive inversion recovery CMR images to detect LGE 1 year prior to hospitalization (**left**) and the second day of hospitalization (**right**) are shown. No functional changes were observed on either CMR in both ventricles (i.e., normal LV and RV function). However, LGE was detected (arrow) in the LV myocardium **(right**) as compared to the isolated involvement of RV free wall, a year previously **(left)**, suggesting progression of disease. CMR, cardiac magnetic resonance; ECG, electrocardiogram; LGE, late gadolinium enhancement; LV, left ventricular; RV, right ventricular. Source: (69), obtained with permission.

While skin defects become apparent early in life, diagnostic features of ARVC do not develop until late puberty (54). The earliest observed cardiac abnormality was in a 5-year-old girl with the cutaneous phenotype of Naxos disease, homozygous for the *JUP* pathogenic variant. She presented with 14.000/24 h ventricular ectopics, without any detectable myocardial imaging change while, after a non-cardiac death, gross pathology of the whole heart and regular histology were normal. However, electron microscopy of the myocardium revealed biventricular gap junction remodeling and abnormal immunohistology for both connexin43 and plakoglobin at cell-cell junctions (68). Clinical presentation of ARVC as an episode of acute myocarditis has been highlighted also in a boy with Naxos disease. He had been followed-up since infancy due to the phenotype of WH and PPK, and he was a homozygous carrier of the pathogenic *JUP* variant (69). Newly developed patchy LGE changes on CMR and progression of repolarization changes were documented at the age of 14 years following an episode of chest pain (Figure 7). Therefore, a thorough cardiac investigation should be considered in any child with the phenotype of Naxos disease upon an episode of chest pain to detect potentially emerging myocardial defects or ARVC progression.

Symptoms of right heart failure in Naxos disease commonly appear later in the disease course. Isolated reports in toddlers homozygous or compound heterozygotes for *DSP* variants show that cardiomyopathy is clinically manifest with severe biventricular or predominant LV involvement leading to heart failure (60, 63, 70).

**Figure 8:**
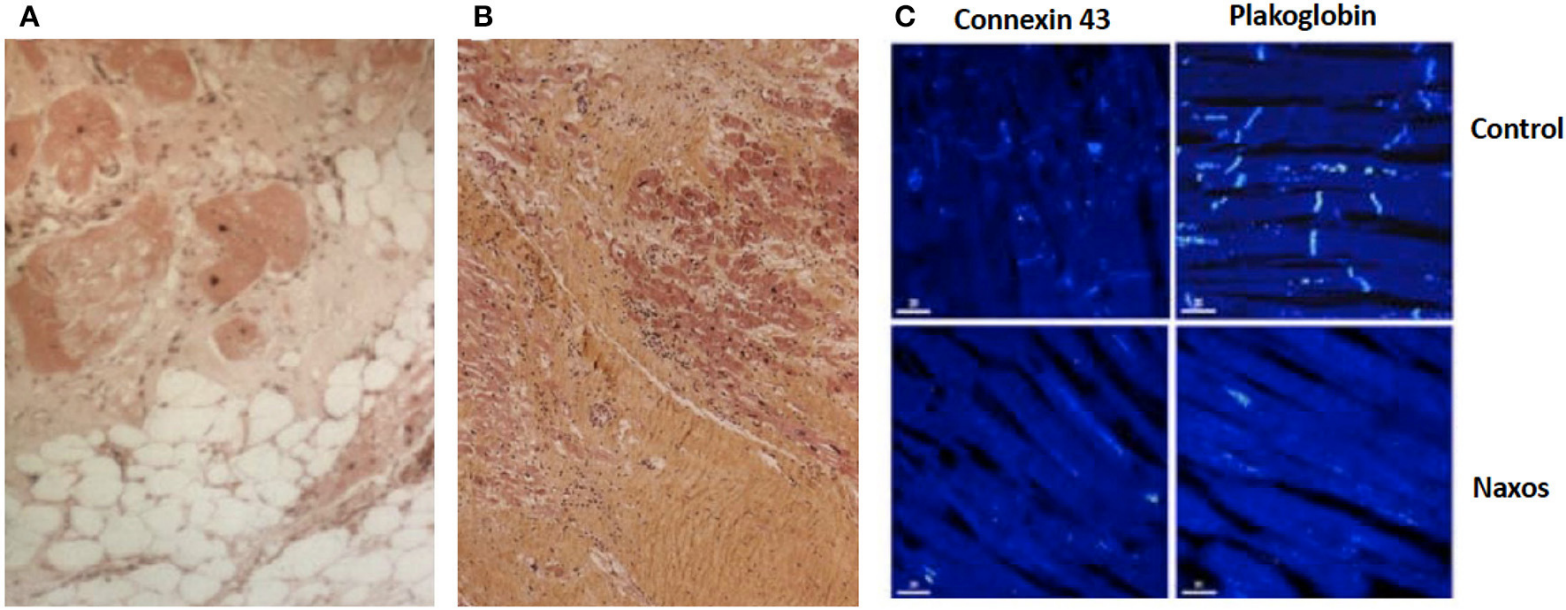
Surgical sample from the anterior wall of the right ventricle of a 19-year-old patient with Naxos disease. Surviving myocardial fibers with some vacuolization, surrounded by fibrosis (haematoxyline-eosine-stained, magnification x 100) **(A)**. Postmortem samples from the left ventricle of a patient with Naxos disease, who died suddenly at the age of 20 years. Myocardial loss with replacement fibrosis and lymphocyte infiltrates (haematoxylin-eosin-stained sections) **(B)**. On confocal microscopy images (same patient) of the left ventricle, there is diminished signal of plakoglobin and connexin 43 at intercellular junctions **(C)**.

Cardiac biopsies performed in patients homozygous for a pathogenic *JUP* variant revealed fibrofatty replacement of RV myocardium. Inflammatory infiltrates were observed particularly when the biopsy was performed at the time of clinical progression (60). In a patient who fulfilled TFC for ARVC diagnosis and died suddenly at the age of 20 years, the RV showed extensive myocardial loss with fibrofatty replacement at subepicardial and medio-mural layers being regionally transmural with aneurysmal formations (68). While the LV appeared unaffected on gross pathology, areas of myocardial loss with fibrofatty substitution or replacement fibrosis with lymphocyte infiltrates were revealed on regular histology (Figures 8A,B). On immunohistology, the signal for plakoglobin and connexin43 were diminished at intercalated disks (Figure 8C).

The rare occurrence of the association of WH and PPK with ARVC illuminated the pathogenesis of the cardiomyopathy (71). Early detection of WH, skin fragility or PPK should warn for ARVC development later in life with variable penetrance. Therefore, a lifelong and targeted cardiac evaluation with 12-lead resting ECG, 24-h Holter monitoring and imaging not only with 2D-echocardiography but also with CMR and LGE is recommended.

### Insights Into Disease Pathogenesis

Important insights into disease pathogenesis can be obtained from patients with Naxos disease (58). In a seminal study by Kaplan et al., electron microscopy of the myocardium revealed smaller and fewer gap junctions in both the RV and LV in a Naxos disease patients, which was accompanied by reduction of connexin43 expression at intercalated disks. Also, the amount of immunofluorescent signal for plakoglobin was significantly reduced at cell-cell junctions in both LV and RV tissue samples. As a result, an ultrastructural mechanism promoting activation delay and re-entry-based arrhythmia was suggested to be related to ARVC (68).

While this provides important clues on ARVC pathogenesis, this might not be the only disease mechanism in young ARVC patients since VT episodes have also been attributed to triggered activity (11, 72). In a recent study of patients diagnosed as CPVT, a *PKP2*-dependent electropathy was suggested as the pathogenetic mechanism of exercise-induced sudden death in those in whom postmortem examination did not reveal any myocardial structural abnormality (73). In several studies, it was shown that decreased desmosomal expression alters the properties of the sodium current and the velocity of the phase 0 upstroke of the action potential (74, 75). In *in vitro* and animal models, this causes significant activation delay, which increases propensity to functional block and promotes arrhythmia susceptibility at a very early disease stage (75, 76).

Also, a cardiomyocyte-specific *PKP2*-knockout mouse model showed that the loss of *PKP2* markedly reduced the transcriptional expression of genes required for intracellular calcium handling (77). Of note, in several published pediatric ARVC series, life-threatening ventricular arrhythmias have mostly been associated with structural abnormalities meeting the imaging TFC (11, 36, 78). Nonetheless, the true “electrical” onset of ARVC in children will be revealed only with prospective clinical cascade family screening.

Evolution of ARVC is a stepwise rather than a linearly progressive process (60). It comes through ‘hot-phases' that may present as myocarditis-like episodes. A potential role of inflammation in ARVC was suggested as early as 1990 (79, 80) and has been increasingly reported since (69, 81–83). In these patients, the presenting symptom might be chest pain, palpitations or presyncope, followed by elevated cardiac troponin levels. Ventricular arrhythmias and repolarization changes on resting 12-lead ECG typically accompany the clinical picture (Figure 7). Abnormal CMR scans with early gadolinium enhancement suggestive of myocardial hyperemia and capillary leak, as well as T2 hyperintensity suggestive of myocardial edema may be detected. Generally, biventricular involvement is revealed.

The myocarditis-like “hot phases”, either as the first clinical presentation or during disease evolution, suggest that an inflammatory process plays a role in disease progression and arrhythmogenesis. In a recent study, monozygotic twins presented with myocarditis at ages 17 and 18 (84). Extensive LV late gadolinium enhancement (LGE) indicative of fibrosis was observed on CMR, and a *DSP* variant was revealed in both patients. In another study, features consistent with myocardial inflammation and severe ventricular arrhythmias were evident in six out of 32 pediatric patients (36). On a molecular level, ARVC is linked to local myocardial production of cytokines and alterations in the balance of circulating inflammatory cytokines.

## Natural History

Three stages in ARVC evolution have been suggested: A “concealed” stage in which the disease is not evident on cardiac screening when only molecular changes can be detected; an “electrical” stage with abnormalities on ECG or Holter monitoring, but a normal or near-normal structure on conventional imaging; and a “structural” stage with expression of the full phenotype. Genotype-phenotype differences and the risk of life-threatening arrhythmias in the concealed stage remain controversial.

The symptomatic disease presentation in children has been reported around the 15th year of life, mostly in series with autosomal dominant ARVC (78). Frequent premature ventricular complexes (PVCs) and episodes of non-sustained VT have been observed as the first electrical manifestations in pediatric ARVC, whilst episodes of syncope or even cardiac arrest have been recorded in young individuals (11). In patients diagnosed under the age of 21 years, cardiac arrest was observed in probands only at a mean age of 15.3 years, and episodes of sustained VT at 16.7 years (11). Electrocardiographic and Holter abnormalities usually precede imaging changes of ventricular myocardium in children (85). Structural abnormalities in children with ARVC are often mild, and involve focal subtricuspid dyskinesia with preserved global function rather than severe RV enlargement (17). When biventricular involvement exists, ARVC is diagnosed in significantly younger age (12.4 ± 5.0 years) as compared to classic ARVC (16.7 ± 2.0 years) (36). Indeed, LV involvement has been related to worse prognosis in children with ARVC (58, 86).

## Cascade Screening

Given the strong familial disease pattern of ARVC, predictive genetic testing and/or cardiac evaluation should be performed in family members of ARVC probands to detect early signs of disease and prevent sudden cardiac death. Indeed, previous studies have shown that approximately one in three family members will develop ARVC (87–89) The yield of screening however varies due to the age-related and incomplete penetrance, and the ARVC disease spectrum can be diverse even among those carrying the same pathogenic variant (85). Ideally, cascade (genetic and/or cardiologic) screening is therefore performed within the confines of a multidisciplinary cardiovascular genetics program that is familiar with the complexity of this disease (2). The following sections will describe the evidence surrounding the questions of genetic as well as cardiac screening for ARVC.

### Cascade Genetic Testing

First, a pedigree should be constructed before genetic testing in the family is started. If a family has multiple affected members, the individual with the youngest presentation and/or most severe disease should be tested first. This is recommended to maximize the chance that if there are multiple P/LP variants in the family they are all identified. As described earlier, a pediatric patient is particularly likely to have more than one P/LP variant.

Second, while a limited ARVC-specific panel is a reasonable choice for first-line genetic testing, increasingly large cardiomyopathy and arrhythmia next generation sequencing panels are used. A recent study has shown using these larger panels (i.e., next generation sequencing of cardiomyopathy and primary arrhythmia-associated genes) do increase detection of clinically informative P/LP variants although at the cost of increased detection of VUSs (90). Nonetheless, as children with ARVC may be particularly likely to have multiple variants, potentially in both desomosomal and non-desmosomal genes, using a larger panel is reasonable to maximize detection of the full genotype.

Third, a recent study by Van Lint et al. of 501 American, Dutch, and German ARVC probands found that P/LP ARVC-associated variants are almost never *de novo* (29). Therefore, if P/LP variant(s) are identified in a child, it is very likely a parent has transmitted the variant and so the parent and also any siblings are at risk. Anticipatory guidance for the family that this is a likely outcome is important.

Finally, children may also be offered genetic testing as part of cascade genetic screening. The age at which pre-symptomatic testing for ARVC is offered varies both within and between nations. Regardless, the decision can be complicated for some families, particularly as detection of a desmosomal variant will likely result in a discussion of the risks and benefits of exercise restriction. A decision-support tool for presymptomatic pediatric ARVC testing for has recently been developed and is publicly available (redcap.link/decision_aid; University of Alberta, accessed 6/20/21).

### Cardiac Screening: “Who” and “When” to Screen for ARVC

**Figure 9:**
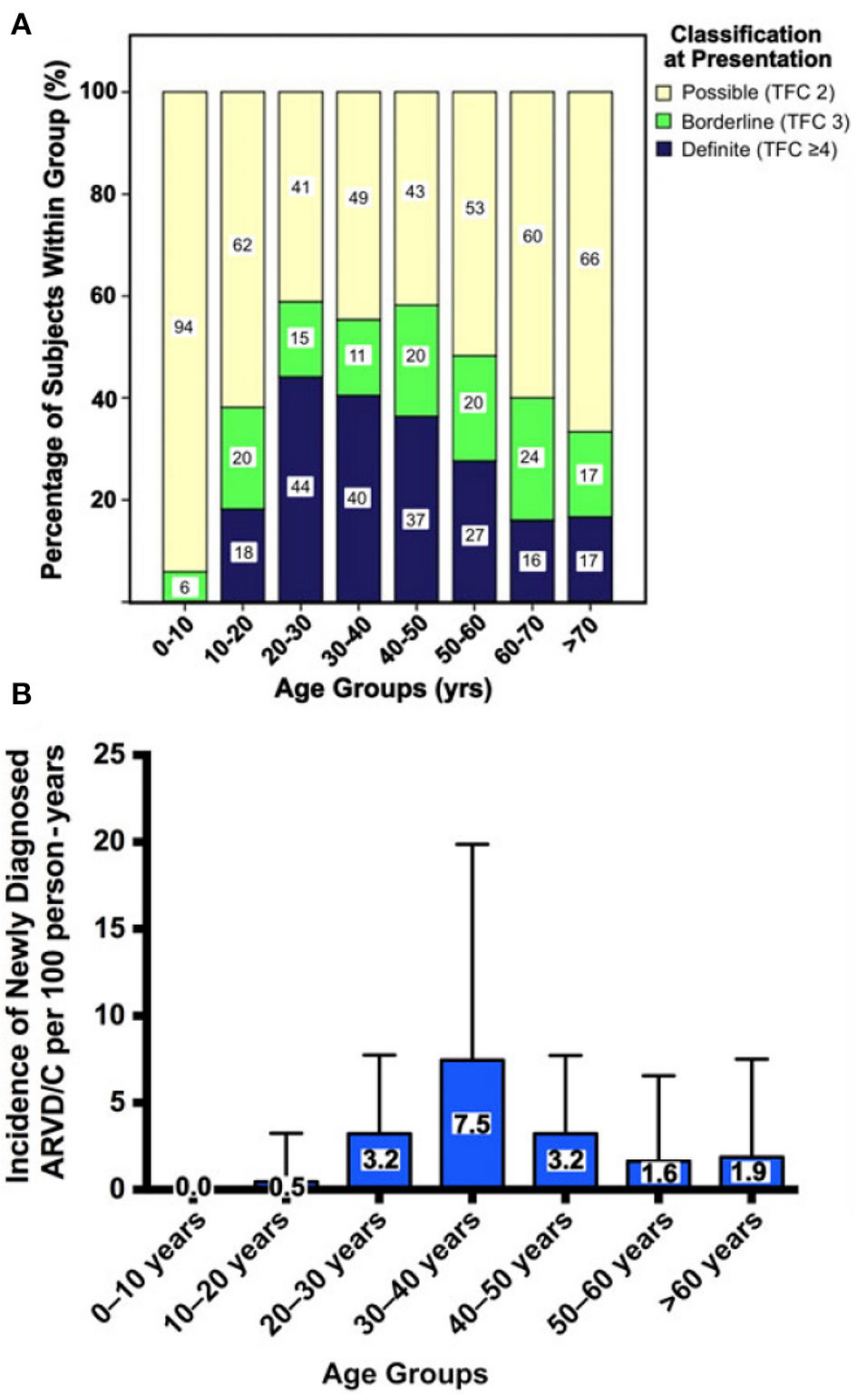
Age-related penetrance of ARVC diagnoses among 274 family members screened for disease. **(A)** Cross-sectional prevalence of ARVC. All family members are stratified by age at evaluation, where each age group is scaled to 100% to show the proportion of family members with definite, borderline, or possible disease at time of evaluation. **(B)** Incidence of newly diagnosed ARVC per 100 person-years during prospective follow-up. Error bars denote upper limit of 95% confidence interval. Source: (89), obtained with permission.

Several research groups have shown that penetrance of ARVC is age-related: ARVC rarely manifests before the age of 10 years, and disease expression increases throughout life with the majority of pathogenic variant carriers expressing disease after 60 years of age (89). Some have suggested that this may be due to the developmental maturation of the intercalated disk, which completes at approximately 10 years after birth and subsequently requires time to get damaged by external and internal factors to ultimately elicit the ARVC phenotype (91). Consequently, most patients are diagnosed with definite ARVC between the second and fourth decade of life. Likewise, a recent study showed that the incidence of newly diagnosed ARVC follows an inverted U-shaped curve, which peaks in the 30–40 year age groups and is lowest at either end of the disease spectrum (Figure 9) (89).

Two recent studies using the same multicenter cohort of 502 definite ARVC patients described clinical features of patients with early (<18 years) or late (>50 years) ARVC presentation (11, 92). Overall, 15% presented as children or adolescents, of whom one in four presented with sudden cardiac death or resuscitated sudden cardiac arrest. Conversely, 21% of ARVC patients presented after the age of 50 years, often with hemodynamically stable VT. While this suggests that all family members of ARVC patients are at risk of developing possibly lethal consequences of this disease, risk of ARVC among family members is not uniform. In a recent study of 274 first-degree family members of ARVC probands, predictors of ARVC diagnosis included symptoms, being a sibling to the proband, and presence of a pathogenic genetic variant (89). Recognition that symptomatic subjects have increased risk of ARVC is intuitive and should be a red flag in any clinical evaluation. The higher risk of disease in siblings as compared to parents and children may be considered surprising and was not completely explained by correcting for age in this study. This may suggest that other factors such as genetic background or shared environmental influences (e.g., exercise participation) impact disease development among family members. Consistent with a genetic inheritance pattern, pathogenic variant carriers have an increased risk of developing disease. Presence of a pathogenic genetic variant even conferred a 6-fold increased risk of disease in a cohort of 302 relatives from 93 families (87). These studies may help clinicians single out family members who should be more closely followed.

### Cardiac Screening: “How to Screen” for ARVC

As described previously, ARVC is diagnosed by a combination of clinical tests as defined in the 2010 TFC. It should therefore be inferred that a full baseline evaluation including all TFC-prescribed tests is necessary to diagnose (or rule out) ARVC with certainty. However, many research groups have provided evidence that diagnostic yield differs among tests.

ARVC is a progressive disease (93), and therefore screening for disease development should be performed at multiple time points using the most useful combination of tests. Almost uniformly, studies have shown that 12-lead ECG and Holter monitoring are among the most sensitive tests for ARVC evaluation, with abnormal test results approaching 90% in affected patients (16). However, almost all these prior studies were cross-sectional in design, and did not consider progression that may have occurred on these tests. Te Riele et al. recently published a report on longitudinal follow-up in family members of ARVC probands (85). In their study, the authors followed 37 asymptomatic family members over a mean of 4.1 ± 2.3 years of follow-up. Overall, 11 (30%) subjects had evidence of disease progression (defined as the development of a new TFC at last follow-up, that was absent at enrolment) and 5 (14%) family members were diagnosed with definite ARVC at last follow-up. Disease progression was most often observed on 12-lead ECG (14%) or Holter monitor (11%), whereas progression on CMR (3%) was rare. In addition, “electrical” changes on ECG and Holter monitor preceded detectable “structural” changed on CMR, and the presence of both an abnormal “electrical” and “structural” substrate magnified arrhythmic risk (94). Taken together, these studies suggest that screening efforts may be most efficient by focusing on ECG and Holter monitoring, while imaging testing should be reserved for selected cases with symptoms and/or abnormal ECG or Holter monitoring tests. Of note, this does not substitute a full baseline evaluation and regular follow-up as per TFC.

### Recommendations for Screening of Pediatric Family Members

In case of a pediatric family member, cascade screening is complicated by the legal and psychological issues that accompany medical interventions in a healthy minor. As such, the Heart Rhythm Society (HRS) Expert Consensus Statement recommends to start ARVC screening at an age when disease expression is expected, i.e., at 10–12 years (2). In contrast, the Heart Failure Society of America (HFSA) suggests that screening should start at an age of 6 years and should be intensified between the ages of 13–19 years (51). Both guidelines recommend the use of genetic testing as well as cardiac evaluation with an ECG, 24-h Holter monitor, and imaging (echocardiography or CMR). Given the peak of ARVC diagnoses in adolescents and young adults, the onset and interval of screening should be adjusted by age, and possibly several other factors including symptomatology and athletic activity.

## Management of ARVC

### Lifestyle/Exercise

In ARVC patients, frequent high-intensity aerobic exercise has been associated with worse clinical outcomes in patients and increased penetrance in at-risk genotype positive relatives. Data from multiple ARVC cohorts consistently shows that competitive or frequent vigorous intensity endurance exercise is associated with earlier onset, sudden cardiac death presentation, worse survival free from ventricular arrhythmias in follow-up, worse RV and LV structure and function, and increased likelihood of heart failure and transplant (95–99). This pattern holds for both gene elusive patients (100) and those with a P/LP variant (95). Reducing exercise after diagnosis results in lower risk of VT and implantable cardioverter-defibrillator (ICD) therapy in follow-up (95, 101). Therefore, although wording varies, guidelines consistently recommend that patients with a definite ARVC diagnosis refrain from competitive or frequent vigorous/high intensity aerobic exercise. The evidence base for exercise guidance for ARVC patients and published clinical recommendations have been recently comprehensively summarized by Zorzi et al. (102).

Exercise has a similar negative impact on patients with the *TMEM43* p.S358L variant, with recent publications showing athletic individuals had earlier onset of ventricular arrhythmias and a considerably worse survival free from appropriate ICD-therapy in follow-up (103). In contrast, a recent publication of a cohort of patients and family members with *DSP* P/LP variants suggested history of exercise was less common in probands and that there was no association of exercise history with sustained ventricular arrhythmias or RV or LV dysfunction (48). While this study requires replication, particularly since it was not designed to measure the patient's exercise history, it does highlight the potential for genotype-specific exercise guidance in the future. Presently, it is important to recognize that current professional recommendations may not accurately reflect predominantly LV disease often associated with *DSP* and *PLN* variants as there is little data to draw on for these populations.

**Figure 10:**
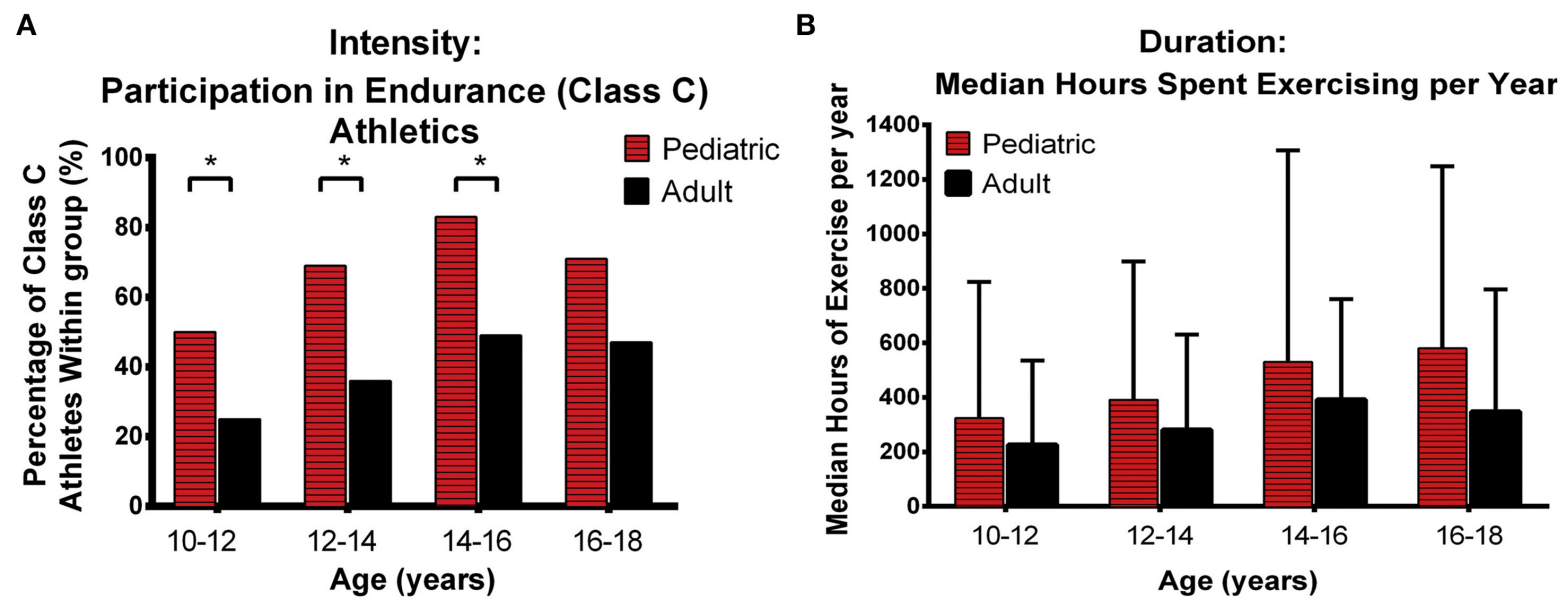
Comparison of exercise intensity and duration during adolescence between patients with pediatric and adult-onset ARVC. Pediatric patients were engaged in higher intensity exercise **(A)** and performed more annual hours of exercise **(B)** during adolescence than arrhythmogenic right ventricular cardiomyopathy patients with Adult onset. Error bars indicate 95% confidence interval. * indicates statistically significant at *p* < 0.05. ARVD/C, arrhythmogenic right ventricular cardiomyopathy. Source: (11), obtained with permission.

There has been limited information specifically focused on the impact of exercise in pediatric patients. Te Riele et al. analyzed exercise participation in 88 patients with P/LP variants (16 pediatric pediatric-onset, 72 adult-onset) who underwent detailed exercise interviews (11). As shown in Figure 10, pediatric patients were significantly more likely than those with adult-onset to have been involved in endurance (Class C) athletics before the age of 18 years (13 of 16 [81%] vs. 39 of 72 [54%]). Specifically, pediatric-onset ARVC was associated with endurance athletics in the 10- to 12-year (*p* = 0.047), 12- to 14-year (*p* = 0.017), and 14- to 16-year (*p* = 0.026) age categories (Figure 10A). In addition, patients with pediatric-onset disease participated in more annual hours of exercise during adolescence compared with patients with adult-onset ARVC, although this did not reach statistical significance (Figure 10B). Also of note, in a recent study of 101 family members of ARVC patients with P/LP variants (mostly in *PKP2*), Wang et al. showed that the difference in exercise between individuals who later developed ARVC and those who remained unaffected was higher in females than in males (104). Girls who had done high-dose exercise in adolescence had the worse survival free from diagnosis. This highlights the importance of “counseling adolescent (and adult) family members with a positive genetic test but who are phenotype negative that competitive or frequent high-intensity exercise is associated with increased likelihood of developing ARVC and ventricular arrhythmias” and engaging in shared decision-making regarding appropriate exercise participation with the family as recommended by recent Heart Rhythm Society professional guidelines developed with multiple international cardiology and genetics societies (2).

### Medical Management and Ablation

Medical therapy plays an important role in the management of patients with ARVC. It must be recognized that there are no prospective randomized or non-randomized clinical trials of any form of medical therapy in ARVC patients of any age. In this section, we will outline the approach to medical therapy employed by the Johns Hopkins ARVC program. It is our impression that this approach is similar to that employed at other high volume ARVC centers.

The cornerstone of medical therapy in ARVC is the use of beta blockers. We believe that the rationale for use of beta blockers is compelling. First, it is well established that beta blockers are the only form of medical therapy that has been shown to reduce the risk of a cardiac arrest in patients with various types of heart disease. Second, beta blockers have been proven to be of benefit in the treatment of patients with various forms of heart failure. Third, a great proportion of cardiac arrests that occur in ARVC patients occur during exercise (95), a situation where catecholamine levels are high. And fourth, Denis and colleagues have demonstrated that a high dose isoproterenol infusion (45 mcg/min) triggers long runs of polymorphic VT in ARVC patients and that this dramatic form of VT responds rapidly to administration of an intravenous beta blocker (105). As such, while conclusive evidence based on (non-)randomized trials is lacking, it is likely that beta-blockers (at least to some degree) prevent ventricular arrhythmias.

Angiotensin converting enzyme (ACE) inhibitors also play an important role in the management of ARVC patients with significant structural disease and RV and/or LV dysfunction. While there are no studies proving their value in ARVC patients, there are many trials demonstrating the critical role of ACE inhibitor therapy in patients with ischemic and non-ischemic forms of dilated cardiomyopathy. We have a low threshold to institute ACE therapy once a beta blocker has been started, assuming the patient tolerates it. Because this is an empiric approach and not proven, we stop the ACE inhibitor if the patient has side effects.

A third form of therapy that we do not employ but you should be aware of is the use of diuretics and nitrates to unload the RV. Fabritz and colleagues demonstrated the value of this approach in a mouse ARVC model (106). More recently, Kalantarian and colleagues published a retrospective analysis of six ARVC patients placed on load-reducing therapy with nitrates and diuretics. The authors reported less RV enlargement during a mean follow-up of 3 years (107). Despite these two studies showing a potential of benefit, we have not employed this form of therapy due to the likelihood of side effects and limitations in the design of these small trials. This is clearly an area for further investigation.

A final type of medical therapy commonly used in ARVC patients are antiarrhythmic drugs. Wichter and colleagues demonstrated the value of sotalol in ARVC patients decades ago (108). In contrast, a more recent study by Marcus showed little value of sotalol, and concluded that amiodarone had greater efficacy (109). Of note, there has been increased interest in the use of flecainide. Ermakov published a small series of combination antiarrhythmic therapy including flecainide (110). Moreover, Cerrone has shown in an animal model that flecainide is of value in a *PKP2* mouse model of ARVC (75). Based on these trials, a prospective randomized clinical trial of flecainide is under way, which is a short-term cross-over study where the endpoint is suppression of PVCs. At present, our preferred antiarrhythmic drug in ARVC patients is flecainide, and if not tolerated or effective we employ sotalol. While we have used amiodarone in the past, we much prefer proceeding to VT ablation rather than start a young person on amiodarone.

Indeed, radiofrequency ablation has gained popularity for ARVC management over the years. While initial studies have indicated that endocardial ablation procedures have a high VT recurrence rate in ARVC patients (111), much better results are obtained with a primary epicardial, or combined endo-epicardial approach (112, 113): in these studies, VT-free survival at 3 years follow-up may even be as high as 85% (114). It should be noted that these ablation results were obtained in high-volume tertiary care centers, and that these results may not pertain to centers that are unfamiliar with ARVC management and its predominantly epicardial substrate (115).

### Arrhythmic Risk Stratification/ICD Implantation

Patients with ARVC have an average annual risk of approximately 10% to develop potentially life-threatening ventricular arrhythmias or sudden cardiac death (116). Of primary concern is sudden cardiac death prevention, for which the only effective treatment is the placement of an ICD. However, this treatment is invasive with inherent complication risk, and can impose physical or psychological burden to the patient. As such, estimating the probability of developing ventricular arrhythmias is pivotal to protect those at high risk, while at the same time limiting interventions among those who are unlikely to derive benefit from their results.

Over the years, many studies have evaluated risk factors for ventricular arrhythmias in ARVC. A full overview of risk factors goes beyond the scope of the present manuscript and can be found elsewhere (116). In short, established predictors of ventricular arrhythmia in ARVC include male sex, younger age at diagnosis, RV systolic dysfunction, prior non-sustained VT, syncope, and high PVC count on Holter monitoring.

**Figure 11:**
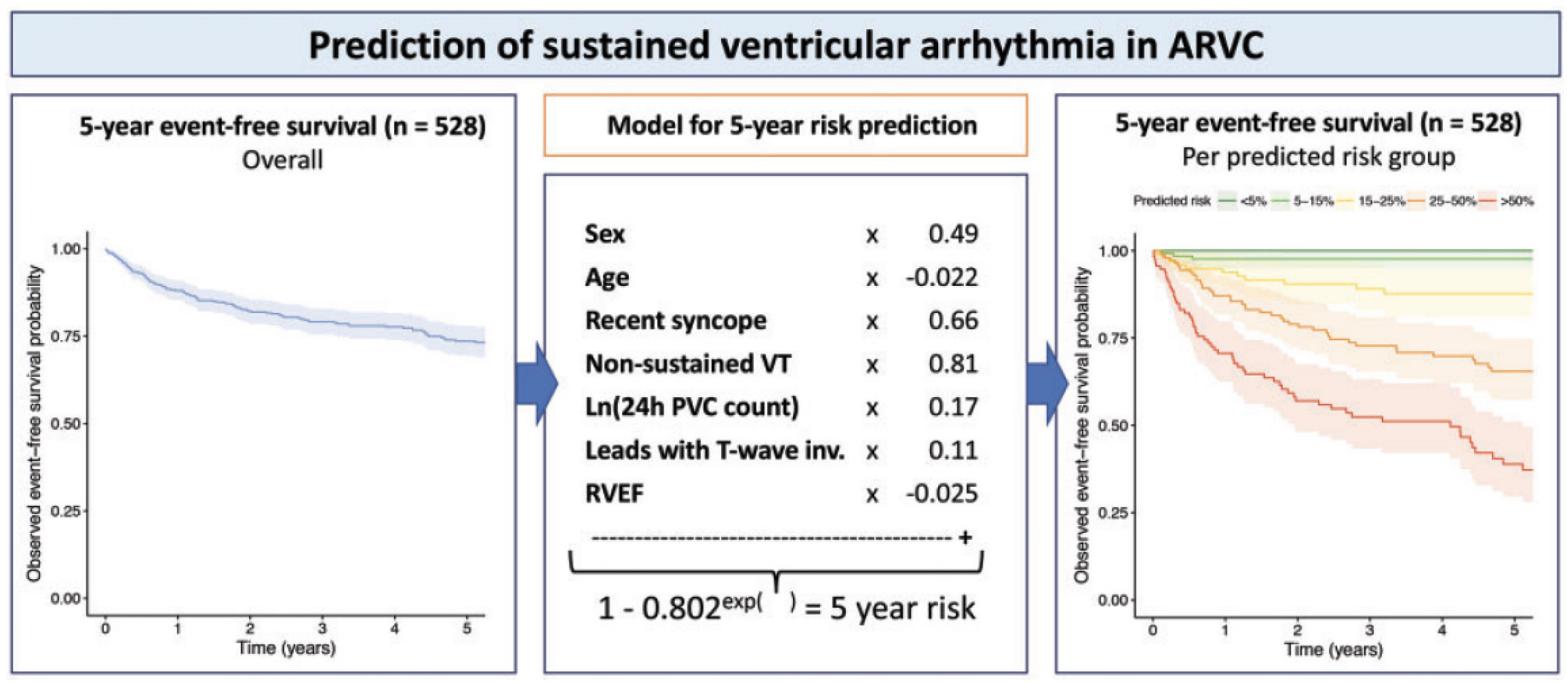
Multivariable risk model to predict occurrence of first sustained ventricular arrhythmia in ARVC patients without prior arrhythmic events. Left panel denotes arrhythmia-free survival in 528 definite ARVC patients in North-America and Europe. Middle panel shows the derivation of a multivariable model for ventricular arrhythmia occurrence, using seven easily available clinical predictors. Right panel shows observed event-free survival stratified by predicted risk in the model. Source: (43), obtained with permission.

Several expert consensus documents recently consolidated the available evidence on arrhythmic risk stratification in ARVC, including the 2015 international task force consensus statement on management of ARVC (117), the 2017 AHA/ACC/HRS guideline for management of ventricular arrhythmias (118), and the 2019 HRS consensus statement on evaluation, risk stratification and management of arrhythmogenic cardiomyopathy (2). While these publications presented a large step forward for clinicians taking care of ARVC patients, several limitations remained: first, these algorithms were based on expert opinion; second, all guidelines were flowchart-based and did not consider the potential interactive effects of combinations of risk factors; and third, translation to absolute risks was lacking. To address these shortcomings, a large transatlantic network of 15 centers in North-America and Europe recently published a multivariable model that enables quantitative individualized prediction of arrhythmic risk in ARVC (Figure 11), which is available for use at www.arvcrisk.com (43, 44). As of today, several studies validated the performance of the risk model in external cohorts, with good to excellent results (119–121). For the purpose of this review, it is however important to highlight that only a minority of study subjects were in the pediatric age range, and future studies should confirm the utility of the risk model in children and adolescents.

## Conclusion

ARVC is an inherited heart disease characterized by fibrofatty infiltration predisposing the patient to ventricular arrhythmias and slowly progressive RV and LV dysfunction. The seminal description of Naxos disease led to the identification of genetic variants in the cardiac desmosome that are associated with ARVC. The disease is typically inherited in an autosomal dominant fashion, with incomplete penetrance and variable expressivity suggesting a strong role for environmental factors. Indeed, exercise exposure and myocardial inflammation have been linked to cardiac disease expression. Given that arrhythmias may occur early in the disease course, early screening and disease detection is of great importance. While there is no definitive cure for ARVC, treatment with beta blockers/antiarrhythmic medication, ACE inhibitors and load reducing therapy may reduce symptoms. Over recent years, several societies provided guidelines for ICD implantation. Future efforts should combine (inter-)national registries to improve our understanding of the clinical characteristics, genetic background, and prognostic factors associated with adverse outcome in ARVC patients at the pediatric age range.

## Data Availability Statement

The original contributions presented in the study are included in the article/supplementary material, further inquiries can be directed to the corresponding author/s.

## Author Contributions

AR, CJ, HC, and AT conceived and designed the research and drafted the manuscript. AR and AT made critical revision of the manuscript for key intellectual content. All authors contributed to the article and approved the submitted version.

## Funding

The Johns Hopkins ARVD/C Program is supported by the Leonie-Wild Foundation, the Leyla Erkan Family Fund for ARVD Research, the Dr. Francis P. Chiramonte Private Foundation, the Dr. Satish, Rupal, and Robin Shah ARVD Fund at Johns Hopkins, the Bogle Foundation, the Healing Hearts Foundation, the Campanella family, the Patrick J. Harrison Family, the Peter French Memorial Foundation, and the Wilmerding Endowments. AR is supported by CVON e-DETECT, the Dutch Heart Foundation (2015T058) and CVON PREDICT Young Talent Program.

## Conflict of Interest

HC receives research support from Boston Scientific Corp. CJ receives salary support from this investigator initiated research grant from Boston Scientific Corp. HC is a consultant for Medtronic Inc., Biosense Webster, Pfizer Inc., and Abbott. CJ is a consultant for Pfizer. The remaining authors declare that the research was conducted in the absence of any commercial or financial relationships that could be construed as a potential conflict of interest.

## Publisher's Note

All claims expressed in this article are solely those of the authors and do not necessarily represent those of their affiliated organizations, or those of the publisher, the editors and the reviewers. Any product that may be evaluated in this article, or claim that may be made by its manufacturer, is not guaranteed or endorsed by the publisher.

## References

[1] Elliott PM Anastasakis A Asimaki A Basso C Bauce B Brooke MA . Definition and treatment of arrhythmogenic cardiomyopathy: an updated expert panel report. Eur J Heart Fail. (2019) 21:955–64. 10.1002/ejhf.1534 31210398 PMC6685753

[2] Towbin JA McKenna WJ Abrams DJ Ackerman MJ Calkins H Darrieux FCC . 2019 HRS expert consensus statement on evaluation, risk stratification, and management of arrhythmogenic cardiomyopathy: Executive summary. Heart Rhythm. (2019) 16:e373–407. 10.1016/j.hrthm.2019.09.019 31676023

[3] Marcus FI Fontaine GH Guiraudon G Frank R Laurenceau JL Malergue C . Right ventricular dysplasia: a report of 24 adult cases. Circulation. (1982) 65:384–98 10.1161/01.CIR.65.2.384 7053899

[4] Sen-Chowdhry S Syrris P Ward D Asimaki A Sevdalis E McKenna WJ. Clinical and genetic characterization of families with arrhythmogenic right ventricular dysplasia/cardiomyopathy provides novel insights into patterns of disease expression. Circulation. (2007) 115:1710–20. 10.1161/CIRCULATIONAHA.106.660241 17372169

[5] Chungsomprasong P Hamilton R Luining W Fatah M Yoo SJ Grosse-Wortmann L. Left ventricular function in children and adolescents with arrhythmogenic right ventricular cardiomyopathy. Am J Cardiol. (2017) 119:778–84. 10.1016/j.amjcard.2016.11.020 28040191

[6] Thiene G Nava A Corrado D Rossi L Pennelli N. Right ventricular cardiomyopathy and sudden death in young people. N Engl J Med. (1988) 318:129–33. 10.1056/NEJM198801213180301 3336399

[7] Beffagna G Zorzi A Pilichou K Perazzolo Marra M Rigato I Corrado D . Arrhythmogenic cardiomyopathy. Eur Heart J. (2020) 41:4457–62. 10.1093/eurheartj/ehaa719 33164038

[8] Thiene G Corrado D Basso C. Arrhythmogenic right ventricular cardiomyopathy/dysplasia. Orphanet J Rare Dis. (2007) 2:45. 10.1186/1750-1172-2-45 18001465 PMC2222049

[9] Protonotarios N Tsatsopoulou A Patsourakos P Alexopoulos D Gezerlis P Simitsis S . Cardiac abnormalities in familial palmoplantar keratosis. Br Heart J. (1986) 56:321–6. 10.1136/hrt.56.4.321 2945574 PMC1236865

[10] Nava A Thiene G Canciani B Scognamiglio R Daliento L Buja G . Familial occurrence of right ventricular dysplasia: a study involving nine families. J Am Coll Cardiol. (1988) 12:1222–8. 10.1016/0735-1097(88)92603-4 3170963

[11] Te Riele A James CA Sawant AC Bhonsale A Groeneweg JA Mast TP . Arrhythmogenic right ventricular dysplasia/cardiomyopathy in the pediatric population: clinical characterization and comparison with adult-onset disease. JACC Clin Electrophysiol. (2015) 1:551–60. 10.1016/j.jacep.2015.08.004 29759408

[12] McKoy G Protonotarios N Crosby A Tsatsopoulou A Anastasakis A Coonar A . Identification of a deletion in plakoglobin in arrhythmogenic right ventricular cardiomyopathy with palmoplantar keratoderma and woolly hair (Naxos disease). Lancet. (2000) 355:2119–24. 10.1016/S0140-6736(00)02379-5 10902626

[13] Patel V Asatryan B Siripanthong B Munroe PB Tiku-Owens A Lopes LR . State of the art review on genetics and precision medicine in arrhythmogenic cardiomyopathy. Int J Mol Sci. (2020) 21:6615. 10.3390/ijms21186615 32927679 PMC7554944

[14] Groeneweg JA Bhonsale A James CA Te Riele AS Dooijes D Tichnell C . Clinical presentation, long-term follow-up, and outcomes of 1001 arrhythmogenic right ventricular dysplasia/cardiomyopathy patients and family members. Circ Cardiovasc Genet. (2015) 8:437–46. 10.1161/circ.130.suppl_2.13189 25820315

[15] Marcus FI McKenna WJ Sherrill D Basso C Bauce B Bluemke DA . Diagnosis of arrhythmogenic right ventricular cardiomyopathy/dysplasia: proposed modification of the task force criteria. Circulation. (2010) 121:1533–41. 10.1161/CIRCULATIONAHA.108.840827 20172911 PMC2860804

[16] Bosman LP Cadrin-Tourigny J Bourfiss M Aliyari Ghasabeh M Sharma A Tichnell C . Diagnosing arrhythmogenic right ventricular cardiomyopathy by 2010 Task Force Criteria: clinical performance and simplified practical implementation. Europace. (2020) 22:787–96. 10.1093/europace/euaa039 32294163 PMC7203633

[17] Te Riele A Marcus FI James CA Murray BA Tichnell C Zimmerman SL . The value of cardiac magnetic resonance imaging in evaluation of pediatric patients for arrhythmogenic right ventricular dysplasia/cardiomyopathy. J Am Coll Cardiol. (2015) 66:873–4. 10.1016/j.jacc.2015.04.082 26271073 PMC4887128

[18] Migliore F Zorzi A Michieli P Perazzolo Marra M Siciliano M Rigato I . Prevalence of cardiomyopathy in Italian asymptomatic children with electrocardiographic T-wave inversion at preparticipation screening. Circulation. (2012) 125:529–38. 10.1161/CIRCULATIONAHA.111.055673 22179535

[19] Platonov PG Calkins H Hauer RN Corrado D Svendsen JH Wichter T . High interobserver variability in the assessment of epsilon waves: Implications for diagnosis of arrhythmogenic right ventricular cardiomyopathy/dysplasia. Heart Rhythm. (2016) 13:208–16. 10.1016/j.hrthm.2015.08.031 26304715

[20] Maceira AM Prasad SK Khan M Pennell DJ. Reference right ventricular systolic and diastolic function normalized to age, gender and body surface area from steady-state free precession cardiovascular magnetic resonance. Eur Heart J. (2006) 27:2879–88. 10.1093/eurheartj/ehl336 17088316

[21] Etoom Y Govindapillai S Hamilton R Manlhiot C Yoo SJ Farhan M . Importance of CMR within the Task Force Criteria for the diagnosis of ARVC in children and adolescents. J Am Coll Cardiol. (2015) 65:987–95. 10.1016/j.jacc.2014.12.041 25766945

[22] Slesnick T Parks WJ Poulik J Al-Haddad E Vickery J Eskarous H . Cardiac magnetic resonance imaging macroscopic fibro-fatty infiltration of the myocardium in pediatric patients with arrhythmogenic right ventricular cardiomyopathy/dysplasia. Fetal Pediatr Pathol. (2020) 39:455–66. 10.1080/15513815.2019.1675108 31625461

[23] Steinmetz M Krause U Lauerer P Konietschke F Aguayo R Ritter CO . Diagnosing ARVC in pediatric patients applying the revised task force criteria: importance of imaging, 12-lead ECG, and genetics. Pediatr Cardiol. (2018) 39:1156–64. 10.1007/s00246-018-1875-y 29754204

[24] Bomma C Rutberg J Tandri H Nasir K Roguin A Tichnell C . Misdiagnosis of arrhythmogenic right ventricular dysplasia/cardiomyopathy. J Cardiovasc Electrophysiol. (2004) 15:300–6. 10.1046/j.1540-8167.2004.03429.x 15030420

[25] Deshpande SR Herman HK Quigley PC Shinnick JK Cundiff CA Caltharp S . Arrhythmogenic right ventricular cardiomyopathy/dysplasia (arvc/d): review of 16 pediatric cases and a proposal of modified pediatric criteria. Pediatr Cardiol. (2016) 37:646–55. 10.1007/s00246-015-1327-x 26743400

[26] Sreetharan S MacIntyre CJ Fatah M Warren AE Wilson GJ Hamilton RM. Clinical utility of endomyocardial biopsies in the diagnosis of arrhythmogenic right ventricular cardiomyopathy in children. Pediatr Res. (2018) 84:552–7. 10.1038/s41390-018-0093-x 29976970

[27] Daliento L Turrini P Nava A Rizzoli G Angelini A Buja G . Arrhythmogenic right ventricular cardiomyopathy in young versus adult patients: similarities and differences. J Am Coll Cardiol. (1995) 25:655–64. 10.1016/0735-1097(94)00433-Q 7860910

[28] Richards S Aziz N Bale S Bick D Das S Gastier-Foster J . Standards and guidelines for the interpretation of sequence variants: a joint consensus recommendation of the American College of Medical Genetics and Genomics and the Association for Molecular Pathology. Genet Med. (2015) 17:405–24. 10.1038/gim.2015.30 25741868 PMC4544753

[29] van Lint FHM Murray B Tichnell C Zwart R Amat N Lekanne Deprez RH . Arrhythmogenic Right Ventricular Cardiomyopathy-Associated Desmosomal Variants Are Rarely De Novo. Circ Genom Precis Med. (2019) 12:e002467. 10.1161/CIRCGEN.119.002467 31386562

[30] van der Zwaag PA van Rijsingen IA Asimaki A Jongbloed JD van Veldhuisen DJ Wiesfeld AC . Phospholamban R14del mutation in patients diagnosed with dilated cardiomyopathy or arrhythmogenic right ventricular cardiomyopathy: evidence supporting the concept of arrhythmogenic cardiomyopathy. Eur J Heart Fail. (2012) 14:1199–207. 10.1093/eurjhf/hfs119 22820313 PMC3475434

[31] Hodgkinson KA Connors SP Merner N Haywood A Young TL McKenna WJ . The natural history of a genetic subtype of arrhythmogenic right ventricular cardiomyopathy caused by a p.S358L mutation in TMEM43. Clin Genet. (2013) 83:321–31. 10.1111/j.1399-0004.2012.01919.x 22725725

[32] Bhonsale A Groeneweg JA James CA Dooijes D Tichnell C Jongbloed JD . Impact of genotype on clinical course in arrhythmogenic right ventricular dysplasia/cardiomyopathy-associated mutation carriers. Eur Heart J. (2015) 36:847–55. 10.1093/eurheartj/ehu509 25616645

[33] Castelletti S Vischer AS Syrris P Crotti L Spazzolini C Ghidoni A . Desmoplakin missense and non-missense mutations in arrhythmogenic right ventricular cardiomyopathy: Genotype-phenotype correlation. Int J Cardiol. (2017) 249:268–73. 10.1016/j.ijcard.2017.05.018 28527814

[34] Chen L Rao M Chen X Chen K Ren J Zhang N . A founder homozygous DSG2 variant in East Asia results in ARVC with full penetrance and heart failure phenotype. Int J Cardiol. (2019) 274:263–70. 10.1016/j.ijcard.2018.06.105 30454721

[35] James CA Jongbloed JDH Hershberger RE Morales A Judge DP Syrris P . International evidence based reappraisal of genes associated with arrhythmogenic right ventricular cardiomyopathy using the clinical genome resource framework. Circ Genom Precis Med. (2021) 14:e003273. 10.1161/CIRCGEN.120.003273 33831308 PMC8205996

[36] DeWitt ES Chandler SF Hylind RJ Beausejour Ladouceur V Blume ED VanderPluym C . Phenotypic manifestations of arrhythmogenic cardiomyopathy in children and adolescents. J Am Coll Cardiol. (2019) 74:346–58. 10.1016/j.jacc.2019.05.022 31319917 PMC7261020

[37] Rigato I Bauce B Rampazzo A Zorzi A Pilichou K Mazzotti E . Compound and digenic heterozygosity predicts lifetime arrhythmic outcome and sudden cardiac death in desmosomal gene-related arrhythmogenic right ventricular cardiomyopathy. Circ Cardiovasc Genet. (2013) 6:533–42. 10.1161/CIRCGENETICS.113.000288 24070718

[38] Klauke B Gaertner-Rommel A Schulz U Kassner A Zu Knyphausen E Laser T . High proportion of genetic cases in patients with advanced cardiomyopathy including a novel homozygous Plakophilin 2-gene mutation. PLoS ONE. (2017) 12:e0189489. 10.1371/journal.pone.0189489 29253866 PMC5734774

[39] Chen K Rao M Guo G Duru F Chen L Chen X . Recessive variants in plakophilin-2 contributes to early-onset arrhythmogenic cardiomyopathy with severe heart failure. Europace. (2019) 21:970–7. 10.1093/europace/euz026 30830208

[40] Ramond F Janin A Di Filippo S Chanavat V Chalabreysse L Roux-Buisson N . Homozygous PKP2 deletion associated with neonatal left ventricle noncompaction. Clin Genet. (2017) 91:126–30. 10.1111/cge.12780 27030002

[41] Verhagen JMA van den Born M Kurul S Asimaki A van de Laar I Frohn-Mulder IME . Homozygous Truncating Variant in PKP2 Causes Hypoplastic Left Heart Syndrome. Circ Genom Precis Med. (2018) 11:e002397. 10.1161/CIRCGEN.118.002397 30562116

[42] Moriarty MA Ryan R Lalor P Dockery P Byrnes L Grealy M. Loss of plakophilin 2 disrupts heart development in zebrafish. Int J Dev Biol. (2012) 56:711–8. 10.1387/ijdb.113390mm 23124967

[43] Cadrin-Tourigny J Bosman LP Nozza A Wang W Tadros R Bhonsale A . A new prediction model for ventricular arrhythmias in arrhythmogenic right ventricular cardiomyopathy. Eur Heart J. (2019) 40:1850–8. 10.1093/eurheartj/ehz103 30915475 PMC6568197

[44] Cadrin-Tourigny J Bosman LP Wang W Tadros R Bhonsale A Bourfiss M . Sudden cardiac death prediction in arrhythmogenic right ventricular cardiomyopathy (ARVC): a multinational collaboration. Circ Arrhythm Electrophysiol. (2020) 14:e008509 33296238 10.1161/CIRCEP.120.008509 PMC7834666

[45] Merner ND Hodgkinson KA Haywood AF Connors S French VM Drenckhahn JD . Arrhythmogenic right ventricular cardiomyopathy type 5 is a fully penetrant, lethal arrhythmic disorder caused by a missense mutation in the TMEM43 gene. Am J Hum Genet. (2008) 82:809–21. 10.1016/j.ajhg.2008.01.010 18313022 PMC2427209

[46] Dominguez F Zorio E Jimenez-Jaimez J Salguero-Bodes R Zwart R Gonzalez-Lopez E . Clinical characteristics and determinants of the phenotype in TMEM43 arrhythmogenic right ventricular cardiomyopathy type 5. Heart Rhythm. (2020) 17:945–54. 10.1016/j.hrthm.2020.01.035 32062046

[47] Vischer AS Castelletti S Syrris P McKenna WJ Pantazis A. Heart failure in patients with arrhythmogenic right ventricular cardiomyopathy: Genetic characteristics. Int J Cardiol. (2019) 286:99–103. 10.1016/j.ijcard.2019.01.065 30765282

[48] Smith ED Lakdawala NK Papoutsidakis N Aubert G Mazzanti A McCanta AC . Desmoplakin Cardiomyopathy, a Fibrotic and Inflammatory Form of Cardiomyopathy Distinct From Typical Dilated or Arrhythmogenic Right Ventricular Cardiomyopathy. Circulation. (2020) 141:1872–84. 10.1161/CIRCULATIONAHA.119.044934 32372669 PMC7286080

[49] Bariani R Cipriani A Rizzo S Celeghin R Bueno Marinas M Giorgi B . 'Hot phase' clinical presentation in arrhythmogenic cardiomyopathy. Europace. (2021) 23:907–17. 10.1093/europace/euaa343 33313835 PMC8184227

[50] Scheel PJ 3rd Murray B Tichnell C James CA Tandri H Calkins H . Arrhythmogenic right ventricular cardiomyopathy presenting as clinical myocarditis in women. Am J Cardiol. (2021) 145:128–134. 10.1016/j.amjcard.2020.12.090 33460606

[51] Hershberger RE Givertz MM Ho CY Judge DP Kantor PF McBride KL . Genetic evaluation of cardiomyopathy-a heart failure society of America Practice Guideline. J Card Fail. (2018) 24:281–302. 10.1016/j.cardfail.2018.03.004 29567486 PMC9903357

[52] James CA Syrris P van Tintelen JP Calkins H. The role of genetics in cardiovascular disease: arrhythmogenic cardiomyopathy. Eur Heart J. (2020) 41:1393–400. 10.1093/eurheartj/ehaa141 32191298

[53] Coonar AS Protonotarios N Tsatsopoulou A Needham EW Houlston RS Cliff S . Gene for arrhythmogenic right ventricular cardiomyopathy with diffuse nonepidermolytic palmoplantar keratoderma and woolly hair (Naxos disease) maps to 17q21. Circulation. (1998) 97:2049–58. 10.1161/01.CIR.97.20.2049 9610536

[54] Protonotarios N Tsatsopoulou A. Naxos disease and Carvajal syndrome: cardiocutaneous disorders that highlight the pathogenesis and broaden the spectrum of arrhythmogenic right ventricular cardiomyopathy. Cardiovasc Pathol. (2004) 13:185–94. 10.1016/j.carpath.2004.03.609 15210133

[55] Norgett EE Hatsell SJ Carvajal-Huerta L Cabezas JC Common J Purkis PE . Recessive mutation in desmoplakin disrupts desmoplakin-intermediate filament interactions and causes dilated cardiomyopathy, woolly hair and keratoderma. Hum Mol Genet. (2000) 9:2761–6. 10.1093/hmg/9.18.2761 11063735

[56] Kaplan SR Gard JJ Carvajal-Huerta L Ruiz-Cabezas JC Thiene G Saffitz JE. Structural and molecular pathology of the heart in Carvajal syndrome. Cardiovasc Pathol. (2004) 13:26–32. 10.1016/S1054-8807(03)00107-8 14761782

[57] Hammill WW Fyfe DA Gillette PC Taylor A Dobson RL Thompson RP. Cardiomyopathy with arrhythmias and ectodermal dysplasia: a previously unreported association. Am Heart J. (1988) 115:373–7. 10.1016/0002-8703(88)90484-X 3341172

[58] Protonotarios N Tsatsopoulou A Anastasakis A Sevdalis E McKoy G Stratos K . Genotype-phenotype assessment in autosomal recessive arrhythmogenic right ventricular cardiomyopathy (Naxos disease) caused by a deletion in plakoglobin. J Am Coll Cardiol. (2001) 38:1477–84. 10.1016/S0735-1097(01)01568-6 11691526

[59] Narin N Akcakus M Gunes T Celiker A Baykan A Uzum K . Arrhythmogenic right ventricular cardiomyopathy (Naxos disease): report of a Turkish boy. Pacing Clin Electrophysiol. (2003) 26:2326–9. 10.1111/j.1540-8159.2003.00370.x 14675023

[60] Protonotarios N Tsatsopoulou A. Naxos disease: cardiocutaneous syndrome due to cell adhesion defect. Orphanet J Rare Dis. (2006) 1:4. 10.1186/1750-1172-1-4 16722579 PMC1435994

[61] Polivka L Bodemer C Hadj-Rabia S. Combination of palmoplantar keratoderma and hair shaft anomalies, the warning signal of severe arrhythmogenic cardiomyopathy: a systematic review on genetic desmosomal diseases. J Med Genet. (2016) 53:289–95. 10.1136/jmedgenet-2015-103403 26399581

[62] Maruthappu T Posafalvi A Castelletti S Delaney PJ Syrris P O'Toole EA . Loss-of-function desmoplakin I and II mutations underlie dominant arrhythmogenic cardiomyopathy with a hair and skin phenotype. Br J Dermatol. (2019) 180:1114–22. 10.1111/bjd.17388 30382575 PMC6318013

[63] Pigors M Schwieger-Briel A Cosgarea R Diaconeasa A Bruckner-Tuderman L Fleck T . Desmoplakin mutations with palmoplantar keratoderma, woolly hair and cardiomyopathy. Acta Derm Venereol. (2015) 95:337–40. 10.2340/00015555-1974 25227139

[64] Chalabreysse L Senni F Bruyere P Aime B Ollagnier C Bozio A . A new hypo/oligodontia syndrome: Carvajal/Naxos syndrome secondary to desmoplakin-dominant mutations. J Dent Res. (2011) 90:58–64. 10.1177/0022034510383984 20940358

[65] Alcalai R Metzger S Rosenheck S Meiner V Chajek-Shaul T. A recessive mutation in desmoplakin causes arrhythmogenic right ventricular dysplasia, skin disorder, and woolly hair. J Am Coll Cardiol. (2003) 42:319–27. 10.1016/S0735-1097(03)00628-4 12875771

[66] Jonkman MF Pasmooij AM Pasmans SG van den Berg MP Ter Horst HJ Timmer A . Loss of desmoplakin tail causes lethal acantholytic epidermolysis bullosa. Am J Hum Genet. (2005) 77:653–60. 10.1086/496901 16175511 PMC1275614

[67] Hobbs RP Han SY van der Zwaag PA Bolling MC Jongbloed JD Jonkman MF . Insights from a desmoplakin mutation identified in lethal acantholytic epidermolysis bullosa. J Invest Dermatol. (2010) 130:2680–3. 10.1038/jid.2010.189 20613772 PMC3061313

[68] Kaplan SR Gard JJ Protonotarios N Tsatsopoulou A Spiliopoulou C Anastasakis A . Remodeling of myocyte gap junctions in arrhythmogenic right ventricular cardiomyopathy due to a deletion in plakoglobin (Naxos disease). Heart Rhythm. (2004) 1:3–11. 10.1016/j.hrthm.2004.01.001 15851108

[69] Mavrogeni S Protonotarios N Tsatsopoulou A Papachristou P Sfendouraki E Papadopoulos G. Naxos disease evolution mimicking acute myocarditis: the role of cardiovascular magnetic resonance imaging. Int J Cardiol. (2013) 166:e14–15. 10.1016/j.ijcard.2012.12.078 23336952

[70] Uzumcu A Norgett EE Dindar A Uyguner O Nisli K Kayserili H . Loss of desmoplakin isoform I causes early onset cardiomyopathy and heart failure in a Naxos-like syndrome. J Med Genet. (2006) 43:e5. 10.1136/jmg.2005.032904 16467215 PMC2564645

[71] Sen-Chowdhry S McKenna WJ. When rare illuminates common: how cardiocutaneous syndromes transformed our perspective on arrhythmogenic cardiomyopathy. Cell Commun Adhes. (2014) 21:3–11. 10.3109/15419061.2013.876415 24460197

[72] Philips B Madhavan S James C Tichnell C Murray B Needleman M . High prevalence of catecholamine-facilitated focal ventricular tachycardia in patients with arrhythmogenic right ventricular dysplasia/cardiomyopathy. Circ Arrhythm Electrophysiol. (2013) 6:160–6. 10.1161/CIRCEP.112.975441 23275260

[73] Tester DJ Ackerman JP Giudicessi JR Ackerman NC Cerrone M Delmar M . Plakophilin-2 Truncation Variants in Patients Clinically Diagnosed With Catecholaminergic Polymorphic Ventricular Tachycardia and Decedents With Exercise-Associated Autopsy Negative Sudden Unexplained Death in the Young. JACC Clin Electrophysiol. (2019) 5:120–7. 10.1016/j.jacep.2018.09.010 30678776 PMC6394846

[74] Sato PY Musa H Coombs W Guerrero-Serna G Patino GA Taffet SM . Loss of plakophilin-2 expression leads to decreased sodium current and slower conduction velocity in cultured cardiac myocytes. Circ Res. (2009) 105:523–6. 10.1161/CIRCRESAHA.109.201418 19661460 PMC2742576

[75] Cerrone M Noorman M Lin X Chkourko H Liang FX van der Nagel R . Sodium current deficit and arrhythmogenesis in a murine model of plakophilin-2 haploinsufficiency. Cardiovasc Res. (2012) 95:460–8. 10.1093/cvr/cvs218 22764151 PMC3422082

[76] Cerrone M Lin X Zhang M Agullo-Pascual E Pfenniger A Chkourko Gusky H . Missense mutations in plakophilin-2 cause sodium current deficit and associate with a Brugada syndrome phenotype. Circulation. (2014) 129:1092–103. 10.1161/CIRCULATIONAHA.113.003077 24352520 PMC3954430

[77] Cerrone M Montnach J Lin X Zhao YT Zhang M Agullo-Pascual E . Plakophilin-2 is required for transcription of genes that control calcium cycling and cardiac rhythm. Nat Commun. (2017) 8:106. 10.1038/s41467-017-00127-0 28740174 PMC5524637

[78] Bauce B Rampazzo A Basso C Mazzotti E Rigato I Steriotis A . Clinical phenotype and diagnosis of arrhythmogenic right ventricular cardiomyopathy in pediatric patients carrying desmosomal gene mutations. Heart Rhythm. (2011) 8:1686–95. 10.1016/j.hrthm.2011.06.026 21723241 PMC3205183

[79] Sabel KG Blomstrom-Lundqvist C Olsson SB Enestrom S. Arrhythmogenic right ventricular dysplasia in brother and sister: is it related to myocarditis? Pediatr Cardiol. (1990) 11:113–6. 10.1007/BF02239576 2140890

[80] Pinamonti B Miani D Sinagra G Bussani R Silvestri F Camerini F. Familial right ventricular dysplasia with biventricular involvement and inflammatory infiltration. Heart Muscle Disease Study Group Heart. (1996) 76:66–9. 10.1136/hrt.76.1.66 8774331 PMC484429

[81] Patrianakos AP Protonotarios N Nyktari E Pagonidis K Tsatsopoulou A Parthenakis FI . Arrhythmogenic right ventricular cardiomyopathy/dysplasia and troponin release. Myocarditis or the “hot phase” of the disease? Int J Cardiol. (2012) 157:e26–28. 10.1016/j.ijcard.2011.09.017 21962611

[82] Martins D Ovaert C Khraiche D Boddaert N Bonnet D Raimondi F. Myocardial inflammation detected by cardiac MRI in Arrhythmogenic right ventricular cardiomyopathy: A paediatric case series. Int J Cardiol. (2018) 271:81–6. 10.1016/j.ijcard.2018.05.116 29885824

[83] Protonotarios A Elliott PM. Arrhythmogenic right ventricular cardiomyopathy as a hidden cause of paediatric myocarditis presentation. Int J Cardiol. (2018) 271:113–4. 10.1016/j.ijcard.2018.06.117 29983250

[84] Reichl K Kreykes SE Martin CM Shenoy C. Desmoplakin Variant-Associated Arrhythmogenic Cardiomyopathy Presenting as Acute Myocarditis. Circ Genom Precis Med. (2018) 11:e002373. 10.1161/CIRCGEN.118.002373 30562115 PMC6300059

[85] te Riele AS James CA Rastegar N Bhonsale A Murray B Tichnell C . Yield of serial evaluation in at-risk family members of patients with ARVD/C. J Am Coll Cardiol. (2014) 64:293–301. 10.1016/j.jacc.2014.04.044 25034067 PMC4380221

[86] Pinamonti B Dragos AM Pyxaras SA Merlo M Pivetta A Barbati G . Prognostic predictors in arrhythmogenic right ventricular cardiomyopathy: results from a 10-year registry. Eur Heart J. (2011) 32:1105–13. 10.1093/eurheartj/ehr040 21362707

[87] Cox MG van der Zwaag PA van der Werf C van der Smagt JJ Noorman M Bhuiyan ZA . Arrhythmogenic right ventricular dysplasia/cardiomyopathy: pathogenic desmosome mutations in index-patients predict outcome of family screening: Dutch arrhythmogenic right ventricular dysplasia/cardiomyopathy genotype-phenotype follow-up study. Circulation. (2011) 123:2690–700. 10.1161/CIRCULATIONAHA.110.988287 21606396

[88] Protonotarios N Anastasakis A Antoniades L Chlouverakis G Syrris P Basso C . Arrhythmogenic right ventricular cardiomyopathy/dysplasia on the basis of the revised diagnostic criteria in affected families with desmosomal mutations. Eur Heart J. (2011) 32:1097–104. 10.1093/eurheartj/ehr043 21345848 PMC3086899

[89] te Riele AS James CA Groeneweg JA Sawant AC Kammers K Murray B . Approach to family screening in arrhythmogenic right ventricular dysplasia/cardiomyopathy. Eur Heart J. (2016) 37:755–63. 10.1093/eurheartj/ehv387 26314686

[90] Murray B Tichnell C Tandri H Calkins H van Tintelen JP Judge DP . Influence of panel selection on yield of clinically useful variants in arrhythmogenic right ventricular cardiomyopathy families. Circ Genom Precis Med. (2020) 13:548–50. 10.1161/CIRCGEN.120.003020 32938230

[91] Vreeker A van Stuijvenberg L Hund TJ Mohler PJ Nikkels PG van Veen TA. Assembly of the cardiac intercalated disk during pre- and postnatal development of the human heart. PLoS ONE. (2014) 9:e94722. 10.1371/journal.pone.0094722 24733085 PMC3986238

[92] Bhonsale A Te Riele A Sawant AC Groeneweg JA James CA Murray B . Cardiac phenotype and long-term prognosis of arrhythmogenic right ventricular cardiomyopathy/dysplasia patients with late presentation. Heart Rhythm. (2017) 14:883–91. 10.1016/j.hrthm.2017.02.013 28215569

[93] Mast TP James CA Calkins H Teske AJ Tichnell C Murray B . Evaluation of Structural Progression in Arrhythmogenic Right Ventricular Dysplasia/Cardiomyopathy. JAMA Cardiol. (2017) 2:293–302. 10.1001/jamacardio.2016.5034 28097316

[94] Zorzi A Rigato I Pilichou K Perazzolo Marra M Migliore F Mazzotti E . Phenotypic expression is a prerequisite for malignant arrhythmic events and sudden cardiac death in arrhythmogenic right ventricular cardiomyopathy. Europace. (2016) 18:1086–94. 10.1093/europace/euv205 26138720

[95] James CA Bhonsale A Tichnell C Murray B Russell SD Tandri H . Exercise increases age-related penetrance and arrhythmic risk in arrhythmogenic right ventricular dysplasia/cardiomyopathy-associated desmosomal mutation carriers. J Am Coll Cardiol. (2013) 62:1290–7. 10.1016/j.jacc.2013.06.033 23871885 PMC3809992

[96] Ruwald AC Marcus F Estes NA 3rd Link M McNitt S Polonsky B . Association of competitive and recreational sport participation with cardiac events in patients with arrhythmogenic right ventricular cardiomyopathy: results from the North American multidisciplinary study of arrhythmogenic right ventricular cardiomyopathy. Eur Heart J. (2015) 36:1735–1743. 10.1093/eurheartj/ehv110 25896080 PMC4500847

[97] Gupta R Tichnell C Murray B Rizzo S Te Riele A Tandri H . Comparison of Features of Fatal Versus Nonfatal Cardiac Arrest in Patients With Arrhythmogenic Right Ventricular Dysplasia/Cardiomyopathy. Am J Cardiol. (2017) 120:111–7. 10.1016/j.amjcard.2017.03.251 28506445

[98] Lie OH Dejgaard LA Saberniak J Rootwelt C Stokke MK Edvardsen T . Harmful effects of exercise intensity and exercise duration in patients with arrhythmogenic cardiomyopathy. JACC Clin Electrophysiol. (2018) 4:744–53. 10.1016/j.jacep.2018.01.010 29929667

[99] Lie OH Chivulescu M Rootwelt-Norberg C Ribe M Bogsrud MP Lyseggen E . Left ventricular dysfunction in arrhythmogenic cardiomyopathy: association with exercise exposure, genetic basis, and prognosis. J Am Heart Assoc. (2021) 10:e018680. 10.1161/JAHA.120.018680 33821670 PMC8174162

[100] Sawant AC Bhonsale A te Riele AS Tichnell C Murray B Russell SD . Exercise has a disproportionate role in the pathogenesis of arrhythmogenic right ventricular dysplasia/cardiomyopathy in patients without desmosomal mutations. J Am Heart Assoc. (2014) 3:e001471. 10.1161/JAHA.114.001471 25516436 PMC4338738

[101] Wang W Orgeron G Tichnell C Murray B Crosson J Monfredi O . Impact of exercise restriction on arrhythmic risk among patients with arrhythmogenic right ventricular cardiomyopathy. J Am Heart Assoc. (2018) 7:e008843. 10.1161/JAHA.118.008843 29909402 PMC6220537

[102] Zorzi A Cipriani A Bariani R Pilichou K Corrado D Bauce B. Role of exercise as a modulating factor in arrhythmogenic cardiomyopathy. Curr Cardiol Rep. (2021) 23:57. 10.1007/s11886-021-01489-0 33961139 PMC8105216

[103] Paulin FL Hodgkinson KA MacLaughlan S Stuckless SN Templeton C Shah S . Exercise and arrhythmic risk in TMEM43 p.S358L arrhythmogenic right ventricular cardiomyopathy. Heart Rhythm. (2020) 17:1159–66. 10.1016/j.hrthm.2020.02.028 32120009

[104] Wang W Tichnell C Murray BA Agafonova J Cadrin-Tourigny J Chelko S . Exercise restriction is protective for genotype-positive family members of arrhythmogenic right ventricular cardiomyopathy patients. Europace. (2020) 22:1270–8. 10.1093/europace/euaa105 32572458

[105] Denis A Sacher F Derval N Lim HS Cochet H Shah AJ . Diagnostic value of isoproterenol testing in arrhythmogenic right ventricular cardiomyopathy. Circ Arrhythm Electrophysiol. (2014) 7:590–7. 10.1161/CIRCEP.113.001224 24970294

[106] Fabritz L Hoogendijk MG Scicluna BP van Amersfoorth SC Fortmueller L Wolf S . Load-reducing therapy prevents development of arrhythmogenic right ventricular cardiomyopathy in plakoglobin-deficient mice. J Am Coll Cardiol. (2011) 57:740–50. 10.1016/j.jacc.2010.09.046 21292134

[107] Kalantarian S Vittinghoff E Klein L Scheinman MM. Effect of preload reducing therapy on right ventricular size and function in patients with arrhythmogenic right ventricular cardiomyopathy. Heart Rhythm. (2021) 18:1186–91. 10.1016/j.hrthm.2021.03.018 33722762

[108] Wichter T Borggrefe M Haverkamp W Chen X Breithardt G. Efficacy of antiarrhythmic drugs in patients with arrhythmogenic right ventricular disease. Results in patients with inducible and noninducible ventricular tachycardia. Circulation. (1992) 86:29–37. 10.1161/01.CIR.86.1.29 1617780

[109] Marcus GM Glidden DV Polonsky B Zareba W Smith LM Cannom DS . Efficacy of antiarrhythmic drugs in arrhythmogenic right ventricular cardiomyopathy: a report from the North American ARVC Registry. J Am Coll Cardiol. (2009) 54:609–15. 10.1016/j.jacc.2009.04.052 19660690 PMC2748738

[110] Ermakov S Gerstenfeld EP Svetlichnaya Y Scheinman MM. Use of flecainide in combination antiarrhythmic therapy in patients with arrhythmogenic right ventricular cardiomyopathy. Heart Rhythm. (2017) 14:564–9. 10.1016/j.hrthm.2016.12.010 27939893

[111] Philips B Madhavan S James C Tichnell C Murray B Dalal D . Outcomes of catheter ablation of ventricular tachycardia in arrhythmogenic right ventricular dysplasia/cardiomyopathy. Circ Arrhythm Electrophysiol. (2012) 5:499–505. 10.1161/CIRCEP.111.968677 22492430

[112] Garcia FC Bazan V Zado ES Ren JF Marchlinski FE. Epicardial substrate and outcome with epicardial ablation of ventricular tachycardia in arrhythmogenic right ventricular cardiomyopathy/dysplasia. Circulation. (2009) 120:366–75. 10.1161/CIRCULATIONAHA.108.834903 19620503

[113] Daimee UA Assis FR Murray B Tichnell C James CA Calkins H . Clinical outcomes of catheter ablation of ventricular tachycardia in patients with arrhythmogenic right ventricular cardiomyopathy: Insights from the Johns Hopkins ARVC Program. Heart Rhythm. (2021) 18:1369–76. 10.1016/j.hrthm.2021.04.028 33933674

[114] Bai R Di Biase L Shivkumar K Mohanty P Tung R Santangeli P . Ablation of ventricular arrhythmias in arrhythmogenic right ventricular dysplasia/cardiomyopathy: arrhythmia-free survival after endo-epicardial substrate based mapping and ablation. Circ Arrhythm Electrophysiol. (2011) 4:478–85. 10.1161/CIRCEP.111.963066 21665983

[115] Haqqani HM Tschabrunn CM Betensky BP Lavi N Tzou WS Zado ES . Layered activation of epicardial scar in arrhythmogenic right ventricular dysplasia: possible substrate for confined epicardial circuits. Circ Arrhythm Electrophysiol. (2012) 5:796–803. 10.1161/CIRCEP.111.967935 22634228

[116] Bosman LP Sammani A James CA Cadrin-Tourigny J Calkins H van Tintelen JP . Predicting arrhythmic risk in arrhythmogenic right ventricular cardiomyopathy: A systematic review and meta-analysis. Heart Rhythm. (2018) 15:1097–107. 10.1016/j.hrthm.2018.01.031 29408436

[117] Corrado D Wichter T Link MS Hauer RN Marchlinski FE Anastasakis A . Treatment of arrhythmogenic right ventricular cardiomyopathy/dysplasia: an international task force consensus statement. Circulation. (2015) 132:441–53. 10.1161/CIRCULATIONAHA.115.017944 26216213 PMC4521905

[118] Al-Khatib SM Stevenson WG Ackerman MJ Bryant WJ Callans DJ Curtis AB . 2017 AHA/ACC/HRS guideline for management of patients with ventricular arrhythmias and the prevention of sudden cardiac death: a report of the american college of cardiology/american heart association task force on clinical practice guidelines and the heart rhythm society. J Am Coll Cardiol. (2018) 72:e91–e220. 10.1016/j.jacc.2017.10.053 29097296

[119] Aquaro GD De Luca A Cappelletto C Raimondi F Bianco F Botto N . Comparison of different prediction models for the indication of implanted cardioverter defibrillator in patients with arrhythmogenic right ventricular cardiomyopathy. ESC Heart Fail. (2020) 7:4080–8. 10.1002/ehf2.13019 32965795 PMC7755004

[120] Casella M Gasperetti A Gaetano F Busana M Sommariva E Catto V . Long-term follow-up analysis of a highly characterized arrhythmogenic cardiomyopathy cohort with classical and non-classical phenotypes-a real-world assessment of a novel prediction model: does the subtype really matter. Europace. (2020) 22:797–805. 10.1093/europace/euz352 31942607

[121] Gasperetti A Russo AD Busana M Dessanai M Pizzamiglio F Saguner AM . Novel risk calculator performance in athletes with arrhythmogenic right ventricular cardiomyopathy. Heart Rhythm. (2020) 17:1251–9. 10.1016/j.hrthm.2020.03.007 32200046

